# Targeted Mutagenesis Reveals Noncanonical Genetic Requirements for Carbon Monoxide Oxidation

**DOI:** 10.1111/1462-2920.70449

**Published:** 2026-11-02

**Authors:** R. A. Dawson, M. Hernández

**Affiliations:** ^1^ School of Biological Sciences University of East Anglia Norwich UK; ^2^ School of Biosciences University of Birmingham Birmingham UK

**Keywords:** carbon monoxide, carbon monoxide dehydrogenase, molecular microbiology, *Paraburkholderia*, targeted mutagenesis

## Abstract

Carbon monoxide (CO) oxidation is a widespread microbial energy metabolism catalysed by MoCu CO dehydrogenase (CODH). Of the two phylogenetically distinct forms (form‐I and form‐II), only form‐I CODH is functionally verified. CODH activity requires maturation of the [CuSMoO_2_] active site, linked in the carboxydotroph *Afipia carboxidovorans* OM5 to the genes *coxDEF*. CODH maturation remains untested outside of this system. *Paraburkholderia terrae* COX, a recently isolated carboxydovore, encodes both forms of CODH with a reduced and reorganised *cox* gene cluster. Using targeted mutagenesis, we identify *rcoM*, *coxL*, *coxF*, and *coxG* as essential for CO oxidation, whereas *coxD, coxE*, and *mocA* are non‐essential. Deletion of the accessory *coxDEF* genes produced distinct phenotypes compared to *A. carboxidovorans* OM5, revealing alternative routes for CODH maturation. Both *coxL* paralogs were deleted singly and in double knockouts, providing the first molecular evidence that form‐II CODH does not contribute to CO oxidation in vivo in these groups of bacteria. Together, these findings show that CODH maturation and function are not universally conserved and caution against inferring CO‐oxidising capacity solely from gene presence. *Pb. terrae* COX emerges as a genetically tractable model for elucidating the diversity and evolution of metalloenzyme assembly pathways in bacteria.

## Graphical abstract

Using targeted mutagenesis, this study shows that form II CODH does not contribute to CO oxidation in the model carboxydovore *Paraburkholderia terrae* COX and reveals that canonical maturation pathways are not universally conserved. These findings refine genome‐based predictions of microbial metabolism and improve understanding of metalloenzyme assembly diversity and evolution.

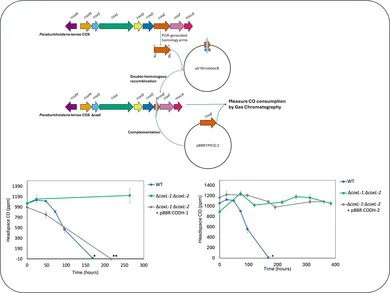

**Abbreviations**
CO: carbon monoxide
CODH: carbon monoxide dehydrogenase
CTP: cytosine triphosphate
MCD: molybdopterin cytosine dinucleotide
MIDAS: metal ion‐dependent adhesion site
Moco: molybdenum cofactor
vWA: von Willebrand factor type A

## 1 Introduction

Oxygen‐tolerant oxidation of carbon monoxide (CO) uses the molybdenum copper enzyme carbon monoxide dehydrogenase (CODH), yielding electrons and CO_2_. This enzyme supports distinct lifestyles in CO‐oxidising microorganisms. Carboxydotrophs gain energy in the form of electrons and carbon through CO_2_ fixation, while carboxydovores only gain energy and release CO_2_ to the environment (King and Weber 2007; Cunliffe 2013). CO‐oxidising bacteria possess a range of CO oxidation‐encoding (*cox*) genes, with the core *coxMSL* genes encoding the heterohexameric CODH (Schubel et al. 1995). In the archetypal carboxydotroph, *Afipia carboxidovorans* OM5, accessory *cox* genes (*coxBCDEFGHIK*) are co‐located with *coxMSL* (Santiago et al. 1999), with all translated gene products except CoxB and CoxK having homologues in other known *cox* gene cluster‐containing bacteria from the Pseudomonadaceae, Comamonadaceae, Nitrobacteraceae, and Mycobacteriaceae (Santiago et al. 1999). In *A. carboxidovorans* OM5, *coxDEFG* have been individually deleted using insertions of a kanamycin resistance cassette, giving valuable insights into the assembly and maturation of the MoCu active site of CODH. Briefly, CoxD is an essential membrane‐bound AAA+ ATPase that incorporates γ‐sulfur, which bridges Cu^I^ and Mo^VI^, converting [MoO_3_] site to [Mo^V^O_2_SH] (Pelzmann et al. 2009; Maisel et al. 2012). CoxF, an XdhC family protein with putative phytase activity (Mickoleit 2017), liberates and binds Cu^II^ from phytate and forms a complex with the membrane‐bound von Willebrand protein CoxE, thereby enabling the reduction of Cu^II^ to Cu^I^ using electrons received from the electron transport chain (Pelzmann et al. 2014). Cu^I^ is coordinated in the active site by two sulfur atoms, one connecting to Mo^VI^ and one to Cys^388^ in the VAYRCSFR active site loop (Dobbek et al. 1999; Meyer et al. 2000; Pelzmann et al. 2014). The accessory *cox* genes *coxBCDEFGHIK* are not universally shared by CO‐oxidising bacteria; these genes are often rearranged or absent when compared to *A. carboxidovorans* OM5 and frequently have low to moderate identity at the deduced amino acid level. *cox* gene clusters have been found containing *coxMSLGDEF* in *Paraburkholderia terrae* COX (Dawson et al. 2025), *xdhC/coxFcoxMSLFDGE* in *Mycobacterium smegmatis* mc^2^155 (CP000480.1, PRJNA92), and *coxMSLFG* in *Thermogemmatispora carboxidivorans* PM5 (JNIM00000000, PRJNA224116) (Figure S1a). *Haliangium ochraceum* DSM 14365 (CP001804, PRJNA28711), a CO‐oxidising δ‐proteobacterium (Quiza et al. 2014), lacks homologues of *coxDEFG* in proximity to the *coxMSL* genes, indicating alternative arrangements of CODH maturation genes in this lineage.

These observations raise an important question: if the *cox* accessory genes are essential to CODH active‐site maturation and enzyme activity, why are they not present in all *cox* gene clusters? Furthermore, when these genes are rearranged or entirely absent, how are the essential functions of CODH active‐site maturation achieved? *Pb. terrae* COX possesses two genes on the *cox* gene cluster which are absent from *A. carboxidovorans* OM5 (Dawson et al. 2025); a putative CO‐sensitive transcriptional regulator *rcoM* (Kerby et al. 2008) and a putative molybdenum cofactor cytidylyltransferase *mocA* (Neumann et al. 2009), suggesting broader differences in CO oxidation between these strains.

While the genetic basis for CO oxidation has been described in *A. carboxidovorans* OM5 (Pelzmann et al. 2009, 2014), mutagenesis experiments in carboxydovores have so far been limited to deletion of the form‐I *coxL* and *coxG* genes in *M. smegmatis* mc^2^155 (Cordero et al. 2019; Kropp et al. 2025). This study therefore aimed to expand our understanding of the molecular genetics of CO oxidation, particularly in carboxydovores. Marker‐free mutations of all accessory *cox* genes and both forms of *coxL* were generated in *Paraburkholderia terrae* COX, a recently isolated carboxydovore from the Pseudomonadota (Dawson et al. 2025). To our knowledge, this study represents the first comprehensive mutagenesis‐based investigation of a CO‐oxidising bacterium in which all major enzymes and transcriptional regulators in the form‐I CODH gene cluster were deleted singly or in combination with other relevant genes. Here, we aimed to determine which accessory *cox* genes are required for CO oxidation in a carboxydovore and how these requirements compared to *A. carboxidovorans* OM5. Additionally, we provide the first molecular evidence that form‐II CODH does not contribute to CO oxidation in vivo in these groups of bacteria.

## 2 Experimental Procedures

### 2.1 Cultivation of *Pb. terrae* COX Strains

Wild‐type *Pb. terrae* COX and all mutant strains (Table S1) were cultivated in VL55 medium (pH 5.5) according to Dawson et al. (2025) using 5 mM sodium pyruvate (Formedium, UK) to support growth, unless stated otherwise. *Pb. terrae* COX form‐I *cox* mutants were supplemented with cysteine, glycine, glutamate, or nicotinic acid according to concentrations available in tryptone and yeast extract (see Supporting Information for full details). CO was added to the headspace of sealed 120 mL vials and measured by gas chromatography as described previously (Dawson et al. 2025) except for the use of an isothermal protocol with the oven temperature held at 100°C for 5 min. CO consumption experiments were performed using three biological replicates per condition. CO consumption by wild‐type *Pb. terrae* COX, serving as a positive control relative to each mutant strain, was conducted once with three biological replicates. As such, it should be acknowledged that sampling effects may influence subsequent analyses of CO consumption data relative to the wild‐type strain. Furthermore, the use of > 1000 ppmv initial concentration of CO precludes environmental conclusions. Full methodological details are provided in Supporting Information.

### 2.2 Plasmid Generation

Plasmids were prepared using restriction digestion and ligation, with *cox* gene‐specific primers designed to include restriction sites (Table S2) to support the assembly of plasmids for mutagenesis in pK18mobsacB and complementation plasmids in pBBR1MCS‐2 (Table S3). Full methodological details are provided in Supporting Information.

### 2.3 Preparation of Competent Cells and Transformation by Electroporation

Electrocompetent *Pb. terrae* COX was prepared according to Choi et al. (2006) with modifications. 100 μL aliquots of electrocompetent cells were transformed with 500 ng of replicable DNA (pBBR1MCS‐2‐based vectors) or 2 μg of non‐replicable DNA (pK18mobsacB‐based vectors) in sterile 2 mm gap cuvettes. Cells were transformed by exponential decay electroporation (2.5 kV, 2.5 μF, 200 Ω) and recovered on SOB agar with 50 μg/mL kanamycin to select for plasmid maintenance. Plasmid acquisition was confirmed by PCR using colony biomass as the template according to Dawson et al. (2025). Full methodological details are provided in Supporting Information.

### 2.4 Preparation of Mutants Using Double‐Homologous Recombination

Single‐recombinants were screened by PCR using colony biomass as the template. All mutants were generated by transformation of wild‐type *Pb. terrae* COX, except for *Pb. terrae* COX ∆*coxL‐1*:∆*coxL‐2* (form‐I and form‐II CODH), which was constructed by transforming *Pb. terrae* COX ∆*coxL‐2* with pK18mobsacB:∆*coxL‐1*. Double‐recombination events were confirmed by PCR using primers specific to the mutated gene (Table S2). Full methodological details are provided in Supporting Information.

### 2.5 Transformation of *Pb. terrae* COX by Conjugation

*Pb. terrae* COX could not be transformed with pK18mobsacB:∆*coxE* by electroporation, although the reason for this was unclear. Conjugation was therefore used as an alternative, with *E. coli* S17‐1 serving as the donor strain to transfer pK18mobsacB:Δ*coxE* into wild‐type *Pb. terrae* COX. Transconjugants were isolated using 50 μg/mL kanamycin for plasmid maintenance and 10 μg/mL gentamicin due to inherent resistance of *Pb. terrae* COX. Full methodological details are provided in Supporting Information.

### 2.6 Bioinformatics Techniques

Protein domains were predicted using BLASTp (Altschul et al. 1990). All alignments of amino acids or nucleotide sequences were conducted using MEGA 11 (Tamura et al. 2021) using ClustalW under standard parameters determined by the software. Full methodological details are provided in Supporting Information.

### 2.7 Statistical Analysis

Statistical analyses were performed in R (version 4.3.2). Statistically significant differences between CO concentrations at the first and final timepoints, used to determine CO consumption over time, were determined by using Welch's 2‐sample *T*‐test in R (package datarium version 0.1.0 (https://rdrr.io/cran/datarium/)). Relative gene expression was analysed separately for each gene and strain using one‐way ANOVA, followed by Tukey's HSD post hoc test. Statistical significance was accepted at *p* < 0.05. Data are presented as the mean ± SD of three biological replicates.

## 3 Results

### 3.1 Updated Genome Sequence Information for *Pb. terrae* COX

Using a combination of short‐read and long‐read sequencing techniques, the genome of *Pb. terrae* COX was closed with 4 circular chromosomes ranging from 1.1 to 4.0 Mbp (Table S4). The *cox* gene cluster (*rcoM_coxMSLGDEF_mocA*), including form‐I CODH, was located on chromosome 1, while the *coxSLM* genes encoding the putative form‐II CODH were located on chromosome 3. Immediately downstream of the form‐II *coxSLM* genes were genes encoding a putative AAA‐family ATPase/MoxR‐like ATPase, a vWA domain‐containing protein, an SRPBCC family protein, and an XdhC family protein (Figure S1b). The deduced amino acid sequences of these proteins shared 49.7%, 37.3%, 28.8%, and 35.1% identity with CoxD, CoxE, CoxG, and CoxF, respectively.

### 3.2 Deletion of the Putative Transcriptional Regulator Gene, *rcoM*

*rcoM* encodes a heme‐containing, CO‐sensitive transcriptional regulator that contains a C‐terminal LytTR DNA‐binding domain and an N‐terminal PAS domain (Kerby et al. 2008). *Pb. terrae* COX ∆*rcoM* failed to consume significant amounts of CO between 0 and 576 h (*p* > 0.05) (Figure 1). Restoration of wild‐type *rcoM* on pBBR1MCS‐2 restored CO oxidation to a similar degree as the wild‐type, as a significant quantity of CO was consumed between 0 and 192 h (*p* ≤ 0.01).

**FIGURE 1:**
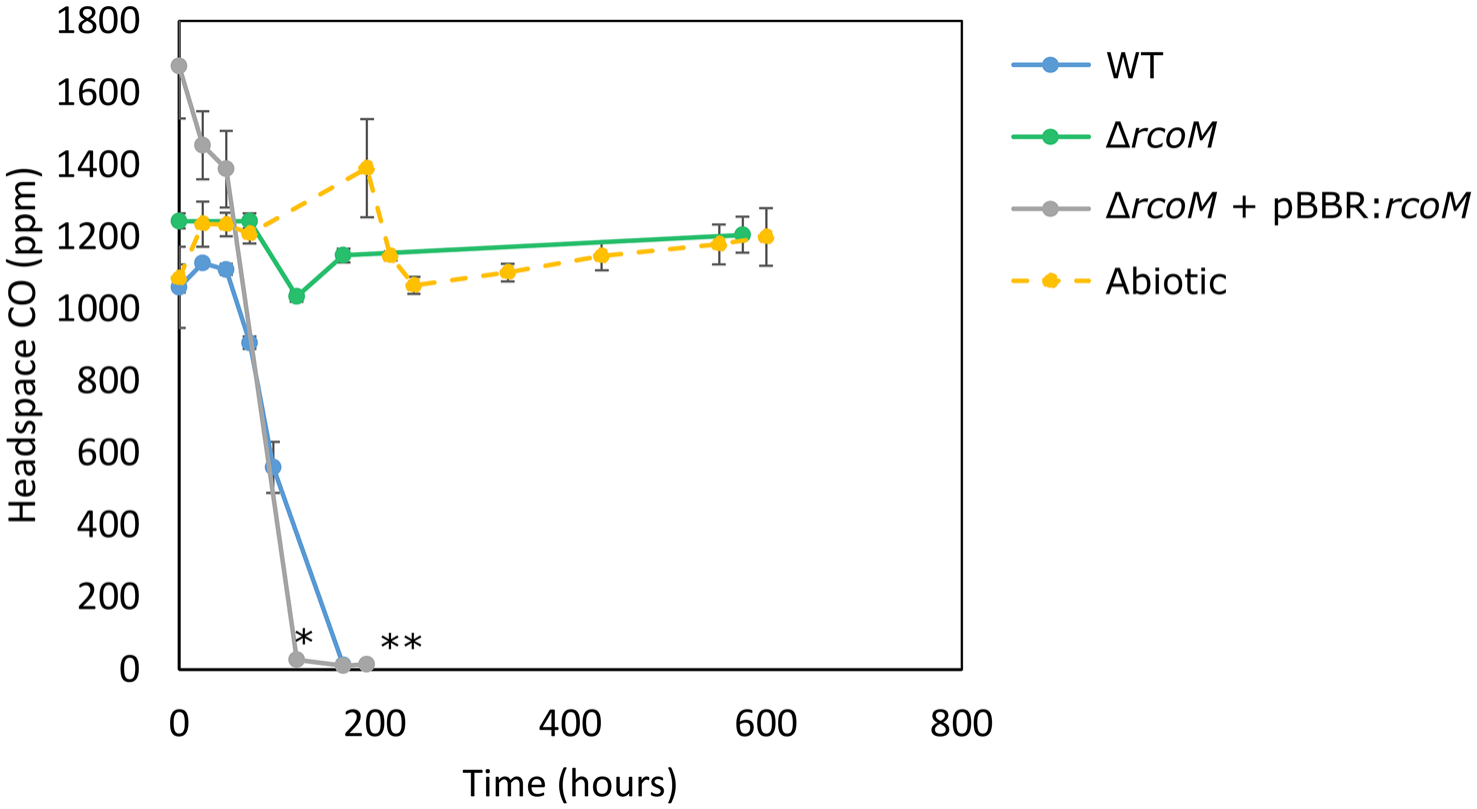
Headspace CO measurements for wild‐type *Pb. terrae* COX, *Pb. terrae* COX ∆*rcoM*, the complemented *Pb. terrae* COX ∆*rcoM* + pBBR:*rcom*, and the abiotic control. Error bars represent the standard error (*n* = 3). (* *= p* ≤ 0.05; ** = *p* ≤ 0.01).

Binding of RcoM upstream of *coxM* requires three discontinuous nucleotide motifs (TTC[AG][TC]GCA) (Dent et al. 2022), termed *d*, *e*, and *f*, respectively, with the former two being highly conserved and the latter being moderately conserved (Dent et al. 2022). The intergenic space between *rcoM* and *coxM* on the genome of *Pb. terrae* COX was aligned against the predicted promoter regions of other *rcoM‐cox* gene clusters; motif *d* adhered to the consensus sequence, but repeats *e* and *f* deviated slightly from the consensus sequence (Figure S2a). Key residues involved in CO‐sensitive transcriptional regulation were identified in the inferred RcoM amino acids from *Pb. terrae* COX (COX‐RcoM) (Figure S2b), including His^74^, Cys^94^, Met^104^, and Met^105^ initially identified in *Paraburkholderia xenovorans* LB400 (Marvin et al. 2008; Bowman et al. 2016).

### 3.3 Large Subunit of CODH

*Pb. terrae* COX ∆*coxL‐1* (form‐I) failed to consume CO between 0 and 360 h (*p* < 0.05) (Figure 2A). The mutant phenotype was complemented by restoring the genes encoding the full wild‐type form‐I CODH (*coxMSL*) to ensure that all CODH‐encoding genes were transcribed and translated together. CODH form‐I in pBBR1MCS‐2 restored the ability of *Pb. terrae* COX ∆*coxL‐1* to consume CO, as the complemented strain consumed a significant amount of CO between 0 and 72 h (*p* ≤ 0.05), while the wild‐type consumed almost all detectable CO within 168 h (Figure 2A). The putative form‐II CODH, encoded by the genes *coxSLM*, was inactivated by generating a markerless mutation in form‐II *coxL*. *Pb. terrae* COX ∆*coxL*‐2 (form‐II) consumed significant quantities of CO within 48 h, compared to the initial timepoint (*p* ≤ 0.001) (Figure 2B), and consumed CO significantly faster than the wild‐type (*p* ≤ 0.01). When aligned against the form‐I CoxL of *A. carboxidovorans* OM5 (OM5‐CoxL), the critical residues for Mo‐ and Cu‐binding in the CODH active site (Gln^240^, Cys^388^, Glu^763^) (Dobbek et al. 1999, 2002) were identified in form‐I CoxL from *Pb. terrae* COX (COX‐CoxL‐1), with only Cys^388^ missing from form‐II CoxL (Figure S3).

**FIGURE 2:**
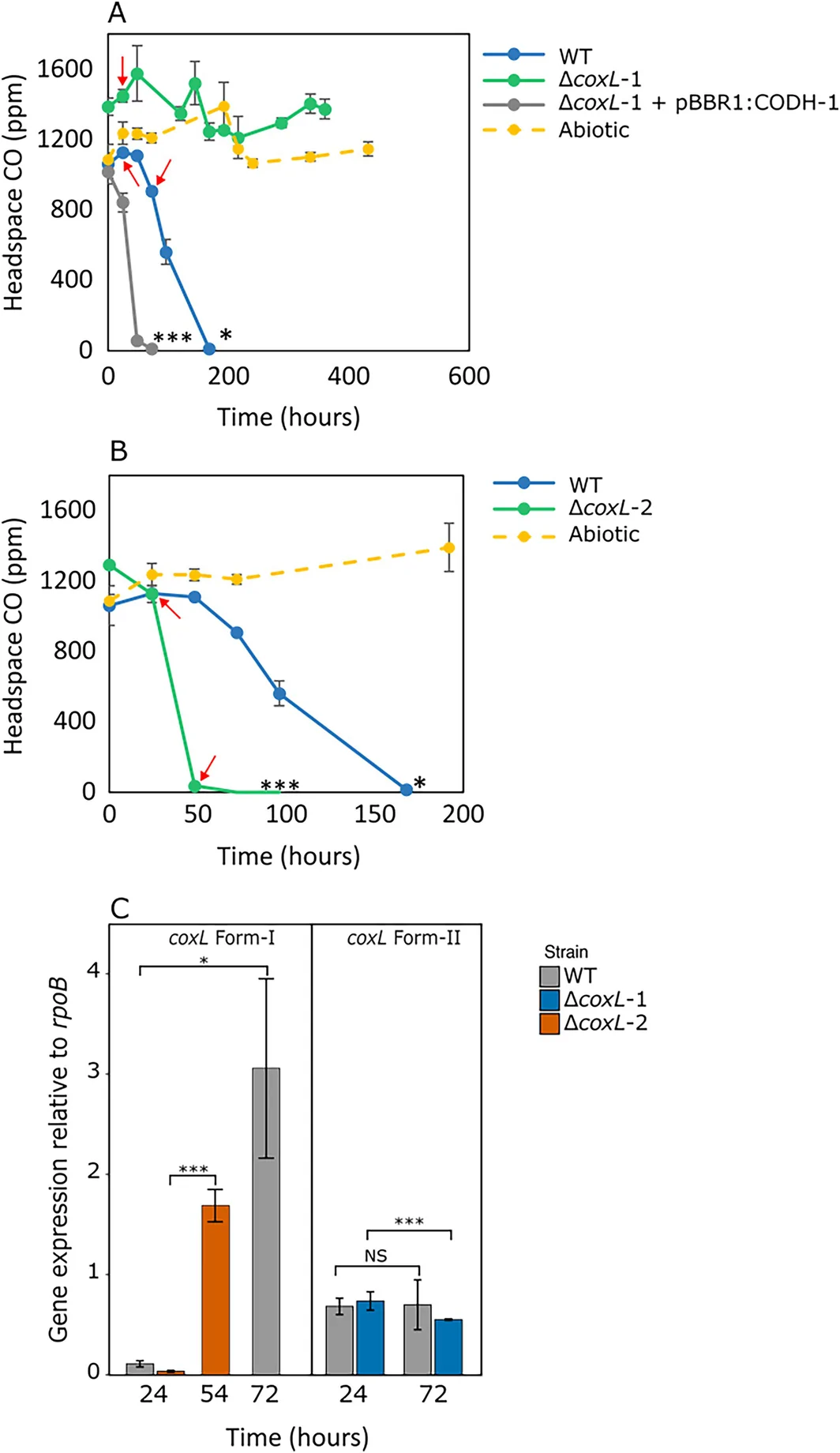
Study of *coxL* form‐I (*coxL‐1*) and form‐II (*coxL‐2*). (A) Headspace CO measurements for wild‐type *Pb. terrae* COX, *Pb. terrae* COX ∆*coxL‐1*, and the complemented *Pb. terrae* COX ∆*coxL* + pBBR:CODH‐1. (B) Consumption of headspace CO by *Pb. terrae* COX ∆*coxL‐2*. (C) Relative expression of *coxL‐1* and *coxL‐2* in wild‐type *P. terrae* COX, Δ*coxL‐1*, and Δ*coxL‐2* strains at different time points, normalised to *rpoB* expression and calculated relative to the corresponding 24 h sample. Statistical analyses were performed separately for each gene and strain across the available time points using one‐way ANOVA followed by Tukey's post hoc test. Bars represent the mean ± SE of three biological replicates (* = *p* ≤ 0.05; *** = *p* ≤ 0.005, NS: Not significant). Red arrows represent the points at which RNA was extracted for RT‐qPCR (see panel c).

*coxL‐1* transcription increased in the wild‐type from 0.11 after 24 h to 3.06 after 72 h (27.7‐fold increase). By comparison, *Pb. terrae* COX ∆*coxL‐2* upregulated *coxL‐1* transcription from 0.05 to 1.69 (47.9‐fold) after 54 h (Figure 2C). *coxL*‐*2* transcription was not notably upregulated in either the wild‐type or ∆*coxL‐1* strains between 24 and 72 h post‐inoculation (Figure 2C), although a low level of constitutive expression was observed.

### 3.4 Role of Form I and Form II CODH in CO Oxidation

Only the deletion of form‐I *coxL* (CODH form‐I) prevented CO consumption by *Pb. terrae* COX (Figure 2A). However, this result did not address the question of whether CODH form‐II was expressed but played no role in CO oxidation in *Pb. terrae* COX, or whether this enzyme was not expressed under the tested conditions. To answer this question, a double‐mutant strain lacking both *coxL*‐*1* (form‐I) and *coxL‐2* (form‐II) was generated. This double‐mutant strain was unable to consume a significant amount of CO between 0 and 384 h (*p* > 0.05) (Figure 3A,B). *Pb. terrae* COX ∆*coxL‐1*:∆*coxL‐2* was transformed with pBBR:CODH‐1 or pBBR:CODH‐2 (Table S3), leading to CODH form‐I (*coxMSL*) and CODH form‐II (*coxSML*) expression at constant but low levels using the pBBR1MCS‐2 promoter (Kovach et al. 1995). CODH form‐I was capable of restoring significant oxidation of CO to ∆*coxL‐1*:∆*coxL‐2* between 0 and 216 h (*p* ≤ 0.01) (Figure 3A). No oxidation of CO was detected from *Pb. terrae* COX ∆*coxL‐1*:∆*coxL‐2* + pBBR:CODH‐2 between 0 and 360 h (Figure 3B).

**FIGURE 3:**
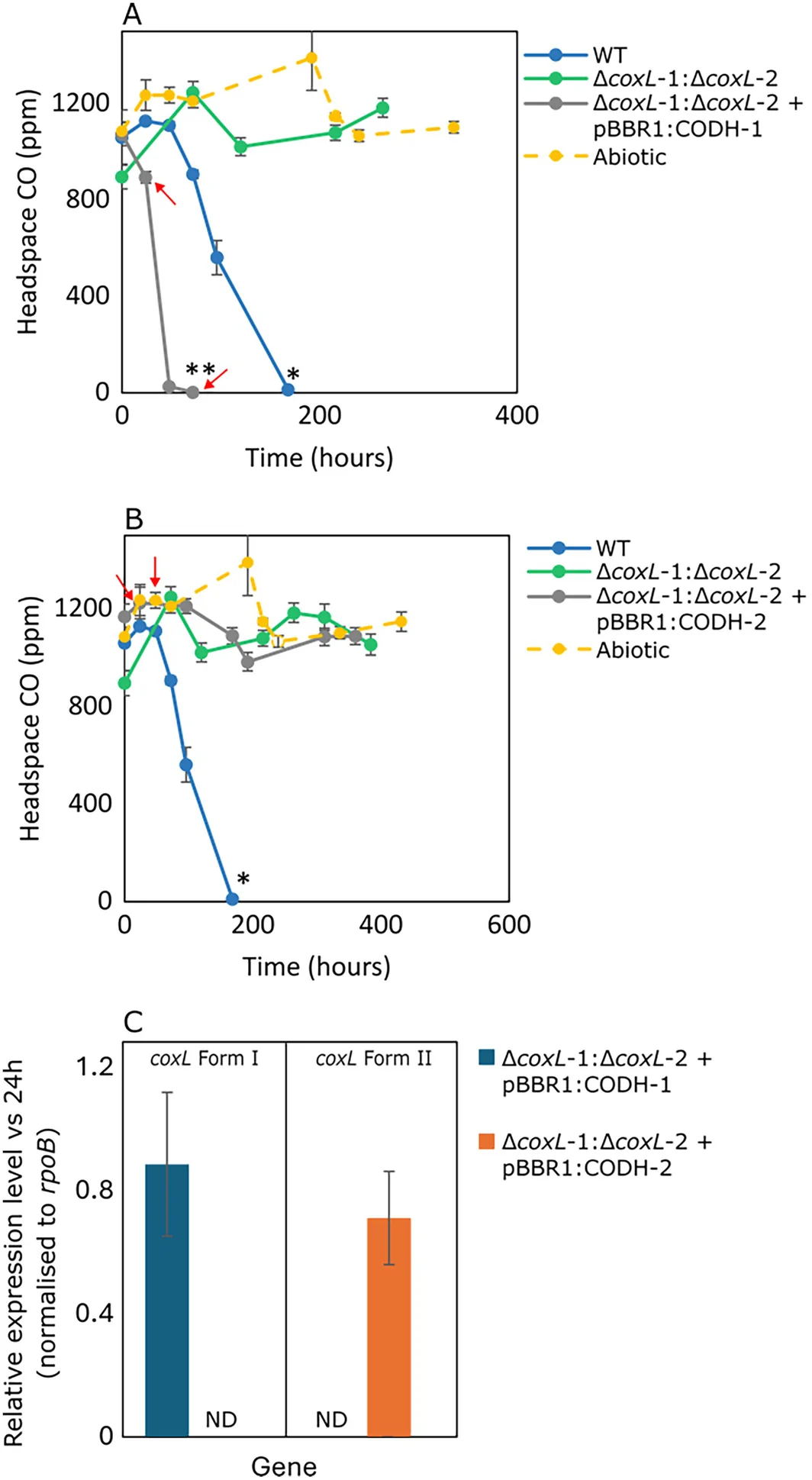
CO consumption by wild‐type *Pb. terrae* COX, *Pb. terrae* COX Δ*coxL*‐1:Δ*coxL*‐2, and the complemented strains (A) *Pb. terrae* COX Δ*coxL*‐1:Δ*coxL*‐2 + pBBR1:CODH‐1 and (B) *Pb. terrae* COX Δ*coxL*‐1:Δ*coxL*‐2 + pBBR1:CODH‐2. (C) Relative expression of *coxL*_1 and *coxL*_2 driven by the pBBR1MCS‐2 promoter in *Pb. terrae* COX Δ*coxL*‐1:Δ*coxL*‐2 carrying pBBR1:CODH‐1 or pBBR1:CODH‐2. Expression at the second timepoint is normalised to the expression level at 24 h of growth for each vector individually. Bars represent the mean ± SE of three biological replicates (* *= p* ≤ 0.05; *** *= p* ≤ 0.005). Red arrows represent the points at which RNA was extracted for RT‐qPCR (see panel c). ND in panel C indicates not determined.

To confirm that CODH‐1 and CODH‐2 were transcribed from the plasmid's de‐repressed *lac* promoter, RT‐qPCR was performed on *Pb. terrae* COX ∆*coxL‐1*:*∆coxL‐2* containing either pBBR1:CODH‐1 or pBBR1:CODH‐2 after 24 h or 72 h post‐inoculation. By studying the relative expression of *coxL‐1* or *coxL‐2*, respectively, it was clear that the genes encoding each form of CODH were transcribed in *Pb. terrae* COX ∆*coxL‐1*:*∆coxL‐2* only when the relevant plasmid was present (Figure 3C).

### 3.5 Deletion of the Predicted AAA+ ATPase Gene, *coxD*

Deletion of *coxD* from *Pb. terrae* COX resulted in a very low rate of CO oxidation when studied in normalised cultures (0.36 ± 0.30 nmol/min/mg) (Figure S4), significantly lower than the wild‐type (*p* ≤ 0.05) and ∆*coxL‐2* strains (*p* ≤ 0.01), and a small but significant amount of CO was consumed between 0 and 528 h (*p* ≤ 0.05) (Figure 4A). This mutant was restored to near wild‐type levels of CO consumption when complemented with *coxD* on pBBR1MCS‐2 (Figure 4A).

**FIGURE 4:**
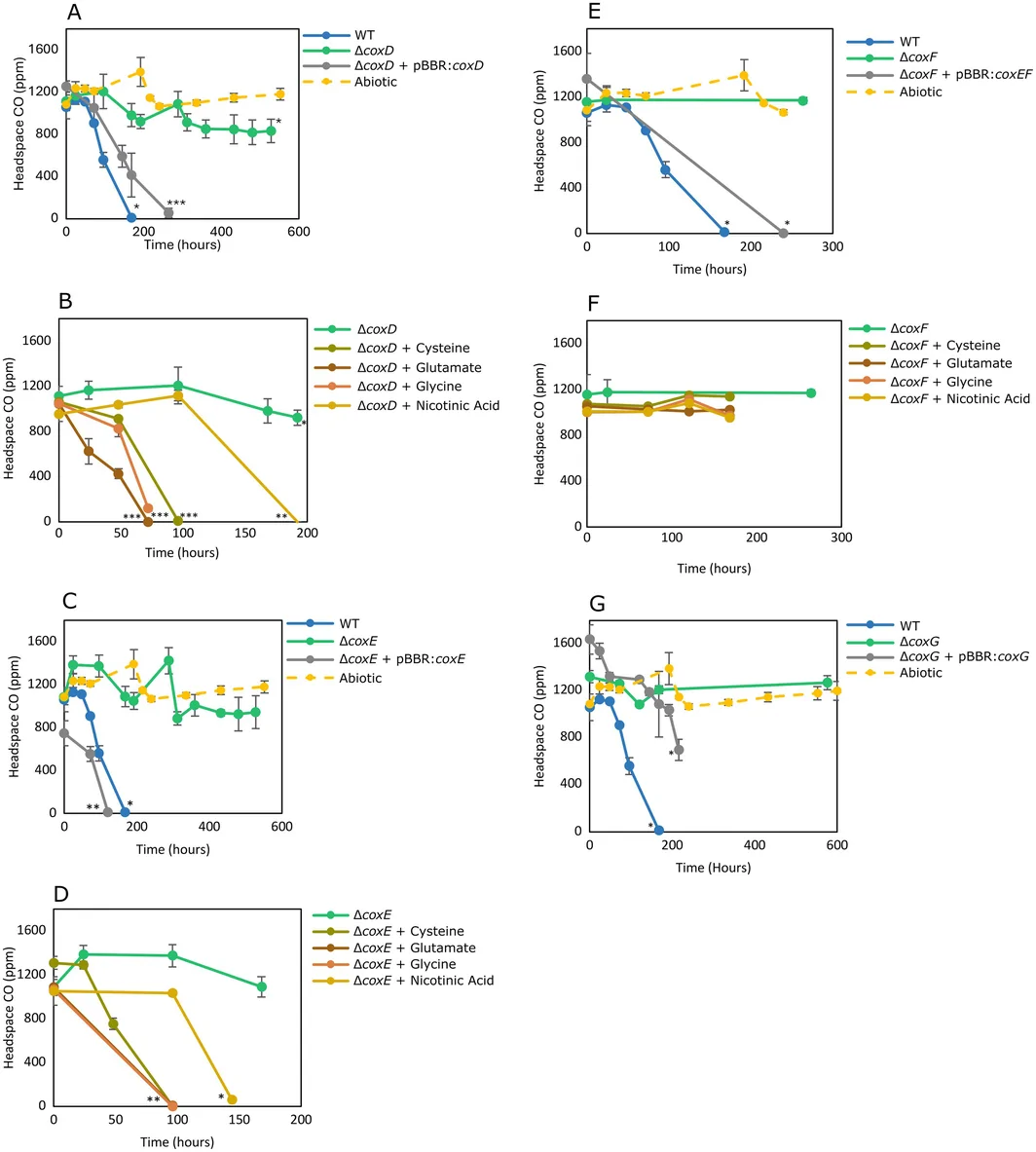
Study of *Pb. terrae* mutants ∆*coxD*, ∆*coxE*, ∆*coxF*, and ∆*coxG*. Error bars represent the standard error (*n* = 3, except where stated). (A) Headspace CO measurements for wild‐type *Pb. terrae* COX, *Pb. terrae* COX ∆*coxD*, the complemented *Pb. terrae* COX ∆*coxD* + pBBR:*coxD*, and the abiotic control. (B) Consumption of CO by *Pb. terrae* COX ∆*coxD* supplemented with cysteine, glutamate, glycine, and nicotinic acid. (C) Headspace CO measurements for wild‐type *Pb. terrae* COX, *Pb. terrae* COX ∆*coxE*, the complemented *Pb. terrae* COX ∆*coxE* + pBBR:*coxE*, and the abiotic control. (D) Consumption of CO by *Pb. terrae* COX ∆*coxE* supplemented with cysteine, glycine, glutamate, and nicotinic acid. (E) Headspace CO measurements for wild‐type *Pb. terrae* COX, *Pb. terrae* COX ∆*coxF*, the complemented *Pb. terrae* COX ∆*coxF* + pBBR:*coxEF*, and the abiotic control. (F) Consumption of CO by *Pb. terrae* COX ∆*coxF* supplemented with cysteine, glycine, glutamate, and nicotinic acid (*n* = 1). (G) Headspace CO measurements for wild‐type *Pb. terrae* COX, *Pb. terrae* COX ∆*coxG*, the complemented *Pb. terrae* COX ∆*coxG* + pBBR:*coxG*, and the abiotic control. (* = *p* ≤ 0.05; ** = *p* ≤ 0.01; *** = *p* ≤ 0.005). For panels a, c, e, and g, blue represents the wild‐type, green represents the mutant, yellow represents the abiotic control, and grey represents the complemented strain.

*Pb. terrae* COX Δ*coxD* consumed significant quantities of CO when supplemented with 0.14 mg/mL cysteine (*p* ≤ 0.001), 3.5 mg/mL glutamate (*p* ≤ 0.001) or 0.98 mg/mL glycine (*p* ≤ 0.001) between 0 and 96 h of growth, whereas supplementation with 0.60 mg/mL nicotinic acid restored CO oxidation with significant loss of CO measured between 0 and 192 h (*p* ≤ 0.01) (Figure 4B).

CoxD from *A. carboxidovorans* OM5 (OM5‐CoxD) possesses putative DEAD‐box RNA helicase motifs, indicative of a role in translational control (Rocak and Linder 2004). CoxD from *Pb. terrae* COX (COX‐CoxD) and all other CoxD proteins studied in this work possessed putative DEAD‐box RNA helicase motifs I, II, and IV, but lacked motifs Ia and V which were found only in CoxD from OM5‐CoxD (Figure S5). Each CoxD protein shared a conserved “arginine finger” and a putative MoxR‐like ATPase domain.

### 3.6 Deletion of the Predicted von Willebrand Family Gene, *coxE*

Deletion of *coxE* resulted in a similar phenotype to *Pb. terrae* COX ∆*coxD*, as CO oxidation was very slow and highly variable in normalised cultures (0.06 ± 1.96 nmol/min/mg) (Figure S4), significantly lower than the ∆*coxL‐2* strain (*p* ≤ 0.01), and a small, insignificant decrease in CO occurred between 0 and 528 h (*p* > 0.05) (Figure 4C). By restoring wild‐type *coxE* on pBBR1MCS‐2, a significant quantity of CO was consumed between 0 and 120 h (*p* ≤ 0.01) (Figure 4C). Compared to the initial CO concentrations (timepoint 0), *Pb. terrae* COX Δ*coxE* consumed significant quantities of CO when supplemented with cysteine (*p* ≤ 0.01), glutamate (*p* ≤ 0.001), and glycine (*p* ≤ 0.001) within 96 h, while supplementation with nicotinic acid restored significant CO oxidation within 144 h (*p* ≤ 0.05) (Figure 4D).

Similar to OM5‐CoxD, CoxE from *A. carboxidovorans* OM5 (OM5‐CoxE) possesses putative DEAD‐box RNA helicase motifs (Pelzmann et al. 2014). However, none of the other CoxE proteins possessed predicted DEAD‐box motifs Ib and III (Figure S6). These CoxE proteins shared the characteristic discontinuous consensus sequence of a metal ion‐dependent adhesion site (MIDAS). The only difference between the MIDAS motifs of COX‐CoxE compared to OM5‐CoxE was an alanine substituted to valine at position 231 and a glycine substituted to arginine at position 233, which was also true for CoxE from most of the other studied CO‐oxidisers (Figure S6). CoxE from *Mycobacterium smegmatis* mc^2^155 (MS‐CoxE) was the most distinct in terms of its predicted MIDAS sites, with numerous substitutions across the discontinuous consensus sequence.

### 3.7 Putative *xdhC* Family Gene, *coxF*

Deletion of *coxF* from *Pb. terrae* COX resulted in a complete cessation of CO oxidation, as no significant decrease in CO was measured between 0 and 264 h (*p* > 0.05, Figure 4E). Similarly, no rate of CO oxidation was detected in normalised cultures (Figure S4). Significant oxidation of CO was measured after restoring the wild‐type *coxEF* genes on pBBR1MCS‐2 (*p* ≤ 0.05) (Figure 4E), but not when wild‐type *coxF* alone was provided (data not shown). When grown in the presence of cysteine (*n* = 1), glutamate (*n* = 1), glycine (*n* = 1), or nicotinic acid (*n* = 1), CO oxidation was not observed after 168 h (Figure 4F).

CoxF from *A. carboxidovorans* OM5 (OM5‐CoxF) possesses two DEAD‐box motifs (III, IV) (Pelzmann et al. 2014), which were absent from all other studied CoxF proteins (Figure S7a). The RGD motif was also lacking from all studied CoxF proteins except for OM5‐CoxF (Figure S7a). Similarly, the histidine acid phosphatase signature possessed by OM5‐CoxF was disrupted in COX‐CoxF by 23 additional amino acids (Figure S7a). OM5‐CoxF and CoxF from *Rhodococcus aggregatum* IAM12617 (IAM‐CoxF) possessed a putative copper‐binding site, which was absent from all other studied CoxF proteins. Instead, CoxF proteins lacking a copper‐binding site (with the exception of CoxF from *M. smegmatis* mc^2^155 (MS‐CoxF)) possessed a putative YHS domain. Alignment of the putative YHS domain of COX‐CoxF against known YHS‐containing proteins revealed that CoxF contained three conserved cysteine residues but possessed an asparagine in place of a conserved aspartate residue (Figure S7b).

### 3.8 Deletion of the Putative SRPBCC Family Gene, *coxG*

CoxG from *Pb. terrae* COX (COX‐CoxG) is a putative SRPBCC family protein, predicted to have a deep binding pocket suited to a hydrophobic ligand. Deletion of *coxG* from *Pb. terrae* COX resulted in a near‐complete cessation of CO oxidation, with a small but insignificant decrease of 50 ppmv CO between 0 and 576 h (*p* > 0.05) (Figure 4G) and a negative rate of −1.70 nmol/min/mg in normalised cultures (Figure S4). This phenotype was alleviated by restoration of wild‐type *coxG* on pBBR1MCS‐2, as significant quantities of CO were consumed between 0 and 216 h (*p* ≤ 0.01). These data confirm that *coxG* is essential to CO oxidation *by Pb. terrae* COX.

Alignment of the deduced amino acids of COX‐CoxG against the other best‐characterised examples of CoxG from *A. carboxidovorans* OM5 (OM5‐CoxG) and *Mycobacterium smegmatis* mc^2^155 (MS‐CoxG) revealed the conservation of one lysine residue in COX‐CoxG and two lysine residues in OM5‐CoxG of the five key lysine residues present in MS‐CoxG (Kropp et al. 2025) (Figure S8).

### 3.9 Deletion of the Putative Molybdenum Cofactor Cytidylyltransferase Gene, *mocA*

The genome of *Pb. terrae* COX possesses a *mocA* gene immediately downstream of the *cox* gene cluster which was absent from the *cox* gene clusters from other representative CO‐oxidising bacteria (Figure S1). The deletion of *mocA* did not affect CO oxidation by *Pb. terrae* COX when compared to the wild‐type (Figure S9a).

Study of the genome of *Pb. terrae* COX revealed putative genes encoding a molybdenum cofactor guanylyltransferase (*mobA*) and a molybdopterin molybdenumtransferase (*moeA*). Additionally, chromosome 1 possessed a second putative *mocA* gene copy, referred to here as *mocA2*, although this shared only 31% identity at the deduced amino acid level with MocA encoded by the *cox* gene cluster. Alignment of MocA and MocA2 amino acids against the functionally verified MocA from *E. coli* revealed the same proposed cytosine triphosphate (CTP) binding motif (Figure S9b).

### 3.10 Putative CODH‐2 Accessory Genes

The predicted gene products of four genes immediately downstream of *coxSLM* were of the same families as CoxD, CoxE, CoxG, and CoxF, respectively, and will henceforth be referred to as CoxD_2, CoxE_2, CoxG_2, and CoxF_2. When aligned with characterised CODH‐1 accessory proteins (CoxD, CoxE, CoxF, CoxG), the putative CoxD_2 lacked OM5‐CoxD‐like DEAD‐box RNA helicase motifs, but possessed a predicted ATP‐binding site and arginine finger (Figure S10a–d). CoxE_2 possessed putative MIDAD motifs but lacked a recognisable DEAD‐box motif Ib. CoxF_2 lacked recognisable DEAD‐box motifs, an RGD motif, histidine acid phosphatase signature, or YHS domain. CoxG_2 similarly shared Lys117 and Lys132, but lacked lysines at positions 50, 81, or 113.

## 4 Discussion

Regulation of CO oxidation is substantially different between characterised strains. *A. carboxidovorans* OM5 possesses two potential transcriptional regulators (*coxC* and *coxH*) (Fuhrmann et al. 2003), with only *coxC* currently confirmed to act as a repressor of *cox* gene transcription (Durante‐Rodríguez et al. 2024), permitting CO oxidation to occur specifically under chemolithoautotrophic conditions (Santiago et al. 1999). *coxC* and *coxH* are absent from many *cox* gene clusters from diverse bacterial lineages (Figure S1). By comparison, the *cox* gene cluster of *Ktedonobacter racemifer* possesses genes immediately upstream of *coxM* which encode a putative histidine kinase (WP_007909435.1, PRJNA27943) and response regulator (WP_007909433.1, PRJNA27943), suggesting the control of CO oxidation by a two‐component system in this bacterium.

*rcoM* has been reported in a limited number of *cox* gene clusters (Kerby et al. 2008; Kerby and Roberts 2012). The *e* and *f* motifs in COX‐RcoM deviated from the (TTC[AG][TC]GCA) consensus sequence, although this was unlikely to have influenced the ability of RcoM to control the transcription of the *cox* gene cluster as the complete absence of CO oxidation by *Pb. terrae* COX ∆*rcoM* confirmed that RcoM is an essential transcriptional activator (Figure 1). Consumption of CO by *Pb. terrae* COX ∆*rcoM* + pBBR:*rcoM* was observed after only 24 h (Figure 1), contrary to wild‐type *Pb. terrae* COX which did not consume significant quantities of CO until stationary phase was reached (Dawson et al. 2025), suggesting the involvement of RcoM in this control of CO oxidation in response to organic carbon starvation.

RcoM is a well‐characterised heme‐containing protein that undergoes a change in oxidation state at the heme bound to His^74^ in the PAS domain, changing from Fe^III^ to Fe^II^, which liberates a cysteine thiolate (Cys^94^). This is replaced by Met^104^, which is then replaced through CO binding to Fe^II^ (Kerby et al. 2008; Marvin et al. 2008; Bowman et al. 2016). The conservation of the His^74^, Cys^94^, and Met^104^ residues in COX‐RcoM indicates that this protein uses the same mechanism of CO‐sensitive transcriptional regulation as the well‐characterised RcoM from *Pb. xenovorans* LB400 (Marvin et al. 2008; Bowman et al. 2016). Met^105^, predicted to stabilise the Fe^III^ heme binding pocket (Bowman et al. 2016), is also present in COX‐RcoM (Figure S2b).

The Mo‐ion in form‐I CoxL from *A. carboxidovorans* OM5 is coordinated by residues Gln^240^, Cys^388^, and Glu^763^, with Cu coordinated to Cys^388^ via the γ‐sulfur atom (Dobbek et al. 1999, 2002). Form‐I CoxL from *Pb. terrae* COX encoded the same essential residues and motifs required for metal ion coordination at the active site and for MCD incorporation (Figure S3), indicating that this protein functions similarly to form‐I CoxL from *A. carboxidovorans* OM5. Combined with the molecular evidence of the deletion of form‐I *coxL* preventing CO oxidation (Figure 2A), this gene clearly encodes the essential active site component of CODH form‐I in *Pb. terrae* COX. The deletion of form‐I *coxL* also prevented CO oxidation by *M. smegmatis* mc^2^155 (Cordero et al. 2019), indicating that CODH form‐I is the determinant of CO oxidation in diverse lineages. Our study presents the novel molecular perspective of a genetically tractable bacterium possessing both forms of CODH, encoded by the canonical *coxMSL* (form‐I) and putative *coxSLM* (form‐II). Form‐II CODH has long been described as “non‐canonical” (King and Weber 2007), and is characterised by a distinct AYRGAGR motif, although some studies have shown that this enzyme can catalyse CO oxidation or even support growth on CO (Lorite et al. 2000; Hogendoorn et al. 2020). The deletion of form‐II *coxL* failed to eliminate the oxidation of CO by *Pb. terrae* COX (Figure 2B), confirming that form‐I CODH is the only active CO‐oxidising enzyme under the tested conditions. In fact, CO consumption was more rapid (Figure S4) and occurred with an earlier onset (Figure 2) when compared to the wild‐type, motivating the harvesting of RNA from CO‐consuming *Pb. terrae* COX ∆*coxL‐2* earlier than the other strains. Furthermore, transcription of *coxL‐2* by wild‐type and ∆*coxL‐1 Pb. terrae* COX was unchanged throughout growth in the presence of CO (Figure 2C), indicating the nonfunction of form‐II CODH in CO oxidation. The increased rate of CO oxidation by *Pb. terrae* COX ∆*coxL‐2* compared to the wild‐type may be due to the amount of available molybdenum cofactor. With both form‐I and form‐II present, wild‐type *Pb. terrae* COX may experience competition for this crucial resource, with form‐II CODH acting as a cofactor sink, while *Pb. terrae* COX ∆*coxL‐2* may experience greater CO oxidation by form‐I CODH due to the loss of this restriction. It should be noted that the use of different secondary timepoints between the wild‐type and ∆*coxL‐2* strains, while warranted by the differing CO oxidation rates, diminishes the ability to form direct comparisons in *coxL‐1* gene expression between these strains. The absence of form‐II *coxL* alleviates the previously observed lag period seen in wild‐type *Pb. terrae* COX before CO consumption is observed. Dawson et al. (2025) demonstrated that CO consumption did not initiate until after the end of growth (stationary phase), corroborated by the increased transcription of form‐I *coxL* between 24 and 72 h (Figure 2C). It is unclear why the deletion of form‐II *coxL* should overcome this inhibition. To our knowledge, this is the first molecular characterisation of form‐II CODH in the context of CO oxidation, although this non‐function has been suggested in other bacteria (Cunliffe 2011). The only previously reported form‐II *coxL* deletion was in *Rubrivivax gelatinosus* (Steunou et al. 2023), where the putative CODH was inferred from the *coxSLM* operon during a study on metal inhibition of xanthine oxidoreductases; however, the ability of this enzyme to oxidise CO was not tested.

A double‐mutant of *Pb. terrae* COX, lacking both form‐I and form‐II CODH, was unable to oxidise CO (Figure 3A,B). This permitted the expression of either form‐I or form‐II CODH from pBBR1MCS‐2 using the plasmid's derepressed *lac* promoter which would constitutively transcribe the cloned genes (Kovach et al. 1995). Constitutive expression was proven by RT‐qPCR of the *coxL‐1* and *coxL‐2* genes (Figure 3C), confirming that *Pb. terrae* COX was incapable of CO oxidation with form‐II *coxL* transcripts present (Figure 3B). This is not direct proof of the presence of a functional form‐II CODH protein; rather, this acts as a strong indication. Corroborating the inability of form‐II CODH to mediate CO oxidation while form‐II *coxL* transcripts are present, the wild‐type and ∆*coxL‐1* strains exhibited similarly constitutive low levels of form‐II *coxL* transcription under the tested conditions. Further molecular characterisations of strains reported to oxidise CO while possessing only form‐II CODH (Lorite et al. 2000; Hogendoorn et al. 2020) would be invaluable, as environmental surveys of *coxL* for CO‐oxidiser abundance and diversity may need to exclude non‐functional form‐II CODH if the inactivity observed in *Pb. terrae* COX is conserved in other bacterial species. The apparently faster expression and assembly of CODH‐1 in *Pb. terrae* COX ∆*coxL‐1* and *Pb. terrae* COX ∆*coxL‐1*:∆*coxL‐2* when compared to the wild‐type (Figures 2A and 3A) raises the question of how CODH‐1 was assembled correctly prior to the normal transcription of *coxDEF*, as the wild‐type apparently does not express active CODH‐1 until 48–72 h post‐inoculation with 5 mM pyruvate (Dawson et al. 2025), while the complemented strains consumed almost all available CO within the first 48 h. Further studies may address this initially by determining the presence or absence of CoxD, CoxE, and CoxF prior to CODH‐1 expression and, potentially, by studying the ability of similar proteins to correctly assemble this Mo‐Cu dehydrogenase.

*coxD* has previously been characterised in *A. carboxidovorans* OM5 (Fuhrmann et al. 2003; Pelzmann et al. 2009; Maisel et al. 2012), demonstrating a role in incorporating a cyanolysable sulfur atom into the CODH active site and allowing development past the inactive [MoO_3_] state. However, OM5‐CoxD also acts as an ATPase with potential roles as a DEAD‐box RNA helicase. Predicted DEAD‐box motifs I (Walker A) and II (Walker B), initially found on OM5‐CoxD (Pelzmann et al. 2014) and indicative of these RNA helicase functions (Rocak and Linder 2004), were found on all other studied CoxD proteins (Figure S5), suggesting that this is a conserved function. Walker A and B together indicate the ability of CoxD to hydrolyse nucleotide triphosphates (Mickoleit 2017), while motif IV is involved in binding phosphate from ATP (Banroques et al. 2011), consistent with the assignment of COX‐CoxD as an AAA+ family ATPase. Motif V, which may be involved in RNA binding (Rocak and Linder 2004), is absent in all other studied CoxD proteins. The deletion of *coxD* in *A. carboxidovorans* OM5 prevented chemolithoautotrophic growth on CO due to OM5‐CODH containing an immature [MoO_3_] active site (Pelzmann et al. 2009), elucidating the essential function of CoxD in CO oxidation. In *Pb. terrae* COX, CoxD was clearly important but not essential to CO oxidation, evidenced by the slow rate of CO oxidation (Figure 4A). Furthermore, the mutant phenotype could be alleviated by supplementary cysteine, nicotinic acid, glycine, and glutamate (Figure 4B), although nicotinic acid restored a slower rate of CO oxidation. Further studies are required to elucidate the active site configuration of CODH in the ∆*coxD* mutant, as continued CO oxidation was not expected if CODH from *Pb. terrae* COX Δ*coxD* possessed a [MoO_3_] centre (Pelzmann et al. 2009).

*coxE* has been similarly characterised in *A. carboxidovorans* OM5 (Fuhrmann et al. 2003; Pelzmann et al. 2014), demonstrating an essential role for this von Willerbrand factor type A (vWA) protein in incorporating copper into the CODH active site. This role may be assisted by CoxF, which was predicted to form a complex with phytate‐derived Cu^2+^. The CoxE‐CoxF:Cu^2+^ complex may form through interaction of the CoxE vWA domain with the RGD motif of CoxF (Campbell and Humphries 2011; Pelzmann et al. 2014; Mickoleit 2017), stabilised by the discontinuous MIDAS motif (Craig et al. 2004) which was conserved across most of the studied CoxE proteins (Figure S6). As a membrane‐bound protein, CoxE was suggested to bring CoxF:Cu^2+^ into proximity with the electron transport chain to reduce copper to Cu^1+^ before delivery to the active site (Pelzmann et al. 2014). The deletion of *coxE* in *Pb. terrae* COX once again failed to completely inhibit CO oxidation (Figure 4C), although the 142 ppmv decrease in CO was deemed insignificant compared to the initial timepoint due to inter‐sample variability (*p* > 0.05, Figure 4C). This indicated that both *coxD* and *coxE* are important to, but not essential for, CO oxidation by this strain. The continued evidence of CO oxidation once again indicated the unexpected presence of mature CODH, while Pelzmann et al. (2014) reported the presence of sulfur but absence of copper from the corresponding ∆*coxE* mutant in *A. carboxidovorans* OM5.

Contextualised with the reported roles of CoxD and CoxE in CODH active site maturation, it is likely that cysteine, glutamate, glycine, and nicotinic acid complement these mutant phenotypes by reconstituting the full [CuSMoO_2_] active site. This would require the provision of the missing cyanolysable sulfur in the case of ∆*coxD*, and Cu^1+^ in the case of ∆*coxE*. We hypothesise that sulfur is transferred from cysteine to the CODH active site to restore CO oxidation in *Pb. terrae* COX ∆*coxD*. Sulfur may be delivered to the CODH active site by a cysteine desulfurase as observed in the *Rhodobacter capsulatus* xanthine dehydrogenase system (Neumann et al. 2007), as an *iscS* homologue is located on chromosome 1 in *Pb. terrae* COX, although this model would assume a previously undescribed role for the XdhC homologue, CoxF (Neumann et al. 2007). The restoration of wild‐type or near wild‐type CO oxidation by glutamate, glycine, and nicotinic acid for *Pb. terrae* COX ∆*coxE* (Figure 4D) has not been reported in another CO oxidising bacterium. The cause for this apparent complementation is currently unclear, but we hypothesise that the copper‐binding ability of each chemical (Bertrán et al. 1999; Selden et al. 2024) is sufficient to complement the copper‐deficient phenotype of CODH in ∆*coxE* mutants, as *A. carboxidovorans* OM5 ∆*coxE* CODH contained sulfur but lacked copper (Pelzmann et al. 2009, 2014). However, certain inconsistencies require further investigation. Firstly, the ability of glutamate, glycine, and nicotinic acid to bind copper does not explain the complementation of the putative sulfur‐deficient ∆*coxD* phenotype (Figure 4B). Secondly, the use of the same active site configurations by ∆*coxD* and ∆*coxE* mutants of *Pb. terrae* COX compared to *A. carboxidovorans* OM5 is unlikely due to the retained CO oxidation activity (Figure 4A,C).

It should be noted that the concentrations of cysteine, glycine, glutamate, and nicotinic acid were non‐environmental, making it unlikely that these organic compounds could perform these supplementations to CO oxidation under native conditions. The lack of reporting of these organic compounds in volcanic soils and sediments prevents their accurate quantification here. However, cysteine is known to be present in the environment, with elevated concentrations reported in the rhizosphere and rapid consumption by soil microbes (Wang et al. 2023), although it is rapidly converted to cystine. Glycine and glutamate concentrations of 0.0005–0.007 mol kg soil^−1^ and 0.0001–0.002 mol kg soil^−1^ were reported in Taibai Mountain (Cao et al. 2016), although their relevance to volcanic environments is unclear. Collectively, the ability of these organic compounds to complement the lost functions of *Pb. terrae* COX ∆*coxD* and *Pb. terrae* COX ∆*coxE* inform our knowledge of the maturation and activity of form‐I CODH, but cannot inform environmental processes without extensive quantification of each compound in situ.

*coxF* was found to be essential to CO oxidation by *Pb. terrae* COX (Figure 4E), a marked difference compared to ∆*coxD* and ∆*coxE* mutants. ∆*coxF*‐deficient mutants of *A. carboxidovorans* OM5 had the same phenotype as the OM5‐∆*coxE* mutant due to a mutual requirement of CoxE and CoxF for their translation, likely due to the predicted DEAD‐box motifs on CoxDEF (Pelzmann et al. 2014). The requirement for pBBR:*coxEF* to complement the ∆*coxF* phenotype in *Pb. terrae* COX (Figure 4E) may indicate a similar translational control. However, several factors suggest that this mutual translation model does not exist in the same way in *Pb. terrae* COX. Firstly, only a small number of DEAD‐box motifs were predicted in CoxD (Figure S5), with the Walker A and Walker B motifs suggesting the ability to bind and hydrolyse ATP and potentially drive DEAD‐box functions (Cordin et al. 2004; Maisel et al. 2012), but no such motifs were found in CoxE (Figure S6) or CoxF (Figure S7). Secondly, mutual translation of COX‐CoxE and COX‐CoxF, resulting in the same CODH phenotype during individual deletions, would imply that the supplementation with cysteine, glutamate, glycine, and nicotinic would work identically on the ∆*coxF* mutant, which was not the case (Figure 4F).

All studied CoxF proteins from predicted and confirmed CO‐oxidising bacteria differed substantially from OM5‐CoxF at the amino acid level (Figure S7). The RGD motif predicted to permit complexing with CoxE (Pelzmann et al. 2014) was only present in OM5‐CoxF. This immediately raises the question of whether complexing of CoxE‐CoxF:Cu^2+^ is a trait of all CO‐oxidising bacteria and what alternatives to RGD‐integrin interactions could be used in *Pb. terrae* COX, or whether an alternative mechanism of Cu incorporation and Cu^1+^ formation is available. Furthermore, the putative “blue copper” copper‐binding site identified in OM5‐CoxF (Pelzmann et al. 2014; Mickoleit 2017) is substantially altered in all other studied CoxF proteins, with the exception of *R. aggregatum* IAM 12617 (Figure S7a). Instead, most CoxF proteins lacking this predicted copper‐binding site—again excluding CoxF from *M. smegmatis* mc^2^155—possessed predicted C‐terminal YHS domains (Figure S7b) which may fulfil a Cu‐binding role (Linder 2015). Alignment of the predicted YHS domain from COX‐CoxF with a database of YHS‐containing proteins (Figure S7b) revealed three conserved cysteine residues, in line with previous research (Linder 2015). However, a conserved aspartate residue was substituted for asparagine in COX‐CoxF (Figure S7b) with unknown consequences for its ability to bind copper. Additionally, the putative role of phytate in providing Cu^2+^ to CoxF (Pelzmann et al. 2014) is brought into question outside of OM5‐CoxF, as the other studied CoxF proteins contained many substituted amino acids within region overlapping the putative histidine acid phosphatase domain (Figure S7a). It therefore remains unclear how CoxF in *Pb. terrae* COX complexes with copper, if or how COX‐CoxF forms a complex with CoxE to mediate Cu^2+^ reduction and, therefore, how Cu^1+^ is delivered to the CODH active site. COX‐CoxF possesses a predicted XdhC‐CoxI family domain, indicating a role in inserting the mature molybdenum cofactor (Moco) into the CODH active site. CoxI may support this process in *A. carboxidovorans* OM5 (Kaufmann et al. 2018), but its absence in *Pb. terrae* COX (Figure S1) and the presence of the homologous XdhC‐CoxI domain on COX‐CoxF may suggest that CoxF fulfils this role in strains naturally lacking CoxI.

CoxG is a membrane‐bound protein which may facilitate the transfer of CO‐derived electrons to the electron transport chain via menaquinone (Pelzmann et al. 2014; Kropp et al. 2025). The deletion of *coxG* resulted in a *Pb. terrae* COX phenotype which was incapable of CO oxidation (Figure 4G), confirming its essential role. This aligned with previous studies in *A. carboxidovorans* OM5 and *M. smegmatis* mc^2^155 (Pelzmann et al. 2014; Kropp et al. 2025): *A. carboxidovorans* OM5 ∆*coxG* exhibited impaired—but not eliminated—chemolithoautotrophic growth on CO, whereas *M. smegmatis* mc^2^155 demonstrated CODH activity only in cell lysates. This was because the lack of CoxG prevented the transfer of electrons derived from CO oxidation via menaquinone to the electron transport system (Kropp et al. 2025). It is therefore likely that *Pb. terrae* COX ∆*coxG* still produces functional CODH but is unable to support CO oxidation without the membrane‐binding CoxG. Kropp et al. (2025) identified five essential lysine residues required by CoxG which generate a positive charge required for association with CODH. When aligned with MS‐CoxG, neither OM5‐CoxG or COX‐CoxG possessed Lys50, Lys81, or Lys113, while only COX‐CoxG lacked Lys132 and all CoxG proteins shared Lys117 (Figure S8). Despite these differences, Kropp et al. (2025) found that structural models of CoxG interacting with CoxMSL were highly consistent, suggesting the ability of different residues to mediate these interactions to the same end and indicating the ability of COX‐CoxG to bind directly to form‐I CODH.

The lengths of time before observing significant decreases in CO by each mutant strain, coupled with the variation in initial CO concentration, made direct comparison of CO consumption rates by each strain difficult. As such, CO uptake rate calculations using normalised inputs of cells from each strain after 72 h aimed to provide a direct comparison of the effects of each mutation. 72 h was chosen as Dawson et al. (2025) previously demonstrated CO oxidation by *Pb. terrae* COX at this time due to the onset of stationary phase. *Pb. terrae* COX ∆*coxL‐2* consumed CO significantly more quickly than the wild‐type strain (Figure S4; *p* ≤ 0.05), consistent with previous data (Figure S2b).

The synthesis of Moco by CO oxidisers, required to produce active CODH, has received little attention, although this process has been better described in the related xanthine dehydrogenase family of enzymes (Neumann et al. 2009). Moco is composed of a Mo ion bound to the enedithiolene group of MCD (Meyer et al. 1993; Dobbek et al. 1999; Gnida et al. 2003). MocA, molybdopterin cytidylyltransferase, catalyses the conversion of molybdenum‐bound molybdopterin and cytidine triphosphate to MCD, an essential part of aldehyde oxidoreductase activity (Neumann et al. 2009). This led to the prediction that MocA would be essential to the assembly of the MCD cofactor required by form‐I CoxL (Tachil and Meyer 2006; Neumann et al. 2009). The deletion of *mocA* failed to alter the ability of *Pb. terrae* COX to oxidise CO, suggesting that MocA is not involved in CO oxidation or, potentially, functional redundancy ensured the continued production of the MCD cofactor. The proposed cytosine binding‐specific aspartate residues on MocA and MocA2 in *Pb. terrae* COX suggest a shared ability to form MCD using CTP (Figure S9b) (Neumann et al. 2009). The MobA protein can likely only generate molybdopterin guanine dinucleotide (Neumann et al. 2009). It is currently unknown whether the chromosomally‐encoded *moeA* is involved in MCD biosynthesis in *Pb. terrae* COX. MocA is present in the *cox* gene clusters of certain β‐ and γ‐proteobacteria, such as *C. ulmosensis* CV2 (JAVCPL000000000, PRJNA1001293), *Pb. xenovorans* LB400 (NC_007951.1, PRJNA224116) and *Alkalilimnicola erhlichii* MLHE‐1 (CP000453.1, PRJNA1001293), but is absent from the *cox* gene clusters of *M. smegmatis* mc^2^155 (CP000480.1, PRJNA92—Actinomycetes), *H. ochraceum* DSM 14365 (GCA_000024805.1, PRJNA28711—Polyangia), *K. racemifer* SOSP1‐21 (GCA_000178855.1, PRJNA27943—Ktedonobacteria), *A. carboxidovorans* OM5 (CP001196.1, PRJNA28805—α‐proteobacteria), and *Labrenzia* sp. CB4 (CP011927.1, PRJNA244670—α‐proteobacteria). While the predicted function of MocA is non‐essential in *Pb. terrae* COX (Figure S9a), it is unclear how the function of MCD biosynthesis is achieved in CO degraders lacking a *mocA* gene homologue. Further study may elucidate which taxa possess MocA and, particularly, the reason for the inclusion of an apparently non‐essential gene involved in MCD biosynthesis across certain *cox* gene clusters.

### 4.1 Putative CODH‐2 Accessory Genes

The presence of genes in *Pb. terrae* COX which encode putative accessory proteins of the same families as CODH‐1 (Figure S10) suggests a similar manner of assembly and maturation as seen in CODH‐1. However, it is unlikely that any of these form‐II accessory proteins are capable of compensating for the loss of their form‐I counterparts under the standard tested conditions, as each deletion of the form‐I accessory proteins resulted in significant disruption or complete cessation of the ability of each strain to oxidise CO (Figure 4). However, it cannot be discounted that the alleviation of form‐I accessory mutant phenotypes at elevated nutrient concentrations were a result of these putative, form‐II accessory genes. As such, further study would greatly benefit from the generation of accessory double‐mutants (e.g., ∆*coxD‐1*: ∆*coxD‐2*) and repeating the nutrient rescue experiments.

While the potential for functional redundancy provided by the accessory form‐II genes cannot be discounted, other bacteria possessing form‐II CODH‐encoding genes do not universally possess the same genes. *C. ulmosensis* CV2 encoded the same putative form‐II CODH accessory proteins immediately downstream of *coxM_2*, while *K. racemifer* possesses two genes immediately downstream of *coxM_2* encoding a putative DoxX family protein (WP_201379026.1) and a leucyl aminopeptidase (WP_201387778.1). Conversely, the noted CODH‐2‐possessing bacterium *Bradyrhizobium diazoefficiens* USDA110 possesses a single gene downstream of *coxM_2* which encodes a putative CaiB/BaiF CoA transferase family protein (BAC45603.1). Two strains of *Kyrpidia spormannii* (COOX1 and FAVT5) were previously shown to grow in the presence of CO despite possessing only CODH‐2 (Hogendoorn et al. 2020), each possess genes encoding a putative AAA family ATPase (WP_100668517.1), similar to CoxD, a vWA domain‐containing protein (WP_170086284.1), similar to CoxE, an XdhC family protein (WP_100668515.1), similar to CoxF, a nucleotidyltransferase family protein (WP_157935520.1), and MoaA (WP_170086281.1), a putative GTP 3′8‐cyclase with a predicted role in molybdenum cofactor assembly (Pang et al. 2025). Collectively, these genome comparisons suggest that certain CODH‐2 enzymes are assembled with the aid of a complement of accessory proteins, similar to CODH‐1.

## 5 Conclusions

CO oxidation by *Pb. terrae* COX is driven by a *cox* gene cluster which is distinct from the previously characterised *cox* cluster from *A. carboxidovorans* OM5. Targeted gene deletions revealed that *rcoM, coxL* (form‐I), *coxF*, and *coxG* are essential to CO oxidation by *Pb. terrae* COX, with *coxD* and *coxE* playing important but non‐essential roles in this process. MocA is not essential to CO oxidation by *Pb. terrae* COX, which we predict to be due to functional redundancy permitting continued MCD production. Key differences in activity between *Pb. terrae* COX mutants lacking *coxD, coxE*, or *coxF* compared to previous examples in *A. carboxidovorans* OM5 have paved the way for in‐depth characterisations of CODH maturation in carboxydovores. This study provides the first molecular evidence for the non‐functionality of form‐II CODH in CO oxidation, suggesting that form‐II *coxL* should not be readily included in surveys of CO oxidiser abundance and diversity in the environment.

## Author Contributions

**R. A. Dawson:** writing – original draft, methodology, writing – review and editing, investigation, visualization, data curation. **M. Hernández:** funding acquisition, writing – review and editing, conceptualization, investigation, visualization, project administration, supervision, resources.

## Funding

This work was supported by the Royal Society, RF\ERE\210050, RF\ERE\231066, DHF\R1\211076.

## Conflicts of Interest

The authors declare no conflicts of interest.

## Supporting information


**Figure S1:** (a) Comparative view of form‐I *cox* gene clusters from distinct CO‐oxidising bacteria. (b) Putative form‐II *cox* gene cluster from *Pb. terrae* COX.
**Figure S2:** (a) MEGA‐aligned amino acid sequences of RcoM proteins, adapted from Dent et al. (2022). Key residues His74, Cys94, and Met104 are highlighted in bold. (b) MEGA‐aligned nucleotides of *rcoM‐coxM* intergenic spaces, adapted from Dent et al. (2022). Consensus sequences d, e, and f are highlighted in bold.
**Figure S3:** MEGA‐aligned amino acid residues from CoxL‐1 from *A. carboxidovorans* OM5 compared to CoxL‐1 and CoxL‐2 from *Pb. terrae* COX. The dinucleotide‐binding motif and Mo‐binding residues are highlighted in bold and positions numbered.
**Figure S4:** Rates of CO uptake by *Pb. terrae* COX strains (nmol/min/mg). * = *p* ≤ 0.05; **= *p* ≤ 0.01.
**Figure S5:** MEGA‐aligned amino acid residues from CoxD proteins from confirmed CO‐oxidising bacteria; *A. carboxidovorans* OM5, *Pb. terrae* COX, *Pb. xenovorans* LB400, *Cupriavidus ulmosensis* CV2, and *M. smegmatis* mc2155. Important motifs and domains are highlighted in bold with uniform colours.
**Figure S6:** MEGA‐aligned amino acid residues from CoxE proteins from confirmed CO‐oxidising bacteria: *A. carboxidovorans* OM5, *Pb. terrae* COX, *Pb. xenovorans* LB400, *Cupriavidus ulmosensis* CV2, and *M. smegmatis* mc2155. Important motifs and domains are highlighted in bold with uniform colours.
**Figure S7:** (a) MEGA‐aligned amino acid residues from CoxF proteins from *A. carboxidovorans* OM5 and *Pb. terrae* COX. Important motifs and domains are highlighted in bold with uniform colours. (b) MEGA‐aligned amino acid residues from the predicted YHS domain of CoxF against YHS domains of reference proteins. This alignment was adapted from Linder (2015).
**Figure S8:** MEGA‐aligned amino acid sequences of CoxG from representative CO‐oxidising bacteria *A. carboxidovorans* OM5, *Pb. terrae* COX, and *M. smegmatis* mc2155. Conserved lysine residues (Kropp et al., 2025) were highlighted in uniform colours.
**Figure S9:** Role of MocA in CO Oxidation by *Pb. terrae* COX. (a) Consumption of CO by *Pb. terrae* COX ∆*mocA* compared to wild‐type *Pb. terrae* COX. Error bars represent the standard error (n = 3). * = *p* ≤ 0.05; *** = *p* ≤ 0.005. (b) Aligned amino acids of MocA and MobA proteins from *Pb. terrae* COX compared to the characterised MocA from *E. coli*. Cytosine‐binding residues were highlighted in uniform colours.
**Figure S10:** Amino acids of putative CODH form II accessory proteins. (a) CoxD_2, (b) CoxE_2, (c) CoxF_2, (d) CoxG_2. Regions aligning with Cox protein motifs are highlighted based on CLUSTALW alignments.
**Table S1:** Strains used in this study.
**Table S2:** List of primers used for mutagenesis and complementation in this study. Highlighted regions denote restriction enzyme recognition sequences.
**Table S3:** Plasmids used in this study.
**Table S4:** Genome information for *Pb. terrae* COX.

## Data Availability

All experimental data have been included in the manuscript and associated Supporting Information. The genome sequence of *Pb. terrae* COX (SAMN36798260) used during all alignments was reported by Dawson et al. (Dawson et al. 2025). The updated genome sequence of *Pb. terrae* COX is available online under the same accession number.
