# Supplementary material for: Targeted Mutagenesis Reveals Noncanonical Genetic Requirements for Carbon Monoxide Oxidation
Source: Environ Microbiol. 2026 Nov 2;28(11):e70449. doi: 10.1111/1462-2920.70449 (PMC13727707; doi:10.1111/1462-2920.70449)
Supplement: Supplementary file 1 [file EMI-28-e70449-s001.pdf]

Supporting Information for:

Targeted mutagenesis reveals noncanonical genetic requirements for carbon monoxide oxidation

Robin A. Dawson[1], Marcela Hernández[1*†]

[1] School of Biological Sciences, University of East Anglia, Norwich, UK, NR4 7TJ.

Running title: Mutagenesis of CO oxidising bacteria

*Corresponding author: Marcela Hernández
[†] Present Address: Marcela Hernández, School of Biosciences, University of Birmingham, Birmingham B15 2TT, UK, m.t.hernandez@bham.ac.uk

This PDF file includes:

Supporting text (Materials and Methods)
Figures S1-S10
Tables S1-S4
SI References

Supporting Information Text
Materials and Methods

Cultivation of *Pb. terrae* COX strains

Wild-type *Pb. terrae* COX and all mutant strains (Table S1) were cultivated in VL55 medium (pH 5.5) according to Dawson *et al.* (2025) using 5 mM sodium pyruvate (Formedium, UK) to support growth, unless stated otherwise. CO was added to the headspace of sealed 120 ml vials at the point of inoculation (T0), with a desired headspace concentration of ~1000 ppmv, and measured by gas chromatography as described previously (Dawson *et al.*, 2025). 100 µl of headspace gas was transferred to an Agilent 7890A gas chromatograph fitted with a HP-Molesieve column (Agilent Technologies; 30 m, 0.53 mm diameter, 25.0 µm film), a nickel catalyst for reduction of CO to CH_4 (350 °C), and a flame ionisation detector (300 °C). A He carrier gas flowed at 4 ml min^{-1}. Standards were prepared in a N_2 atmosphere using 120 ml vials, with CO added from a 10% (v/v) stock to final concentrations of 2,000 ppmv, 1,000 ppmv, 750 ppmv, 500 ppmv, and 250 ppmv. CO consumption experiments were performed using three biological replicates per condition, with 120 ml vials incubated at 30 °C with shaking at 150 rpm. CO consumption by wild-type *Pb. terrae* COX, serving as a positive control relative to each mutant strain, was conducted once with three biological replicates. As such, it should be acknowledged that sampling effects may influence subsequent analyses of CO consumption data relative to the wild-type strain. When supplementing *Pb. terrae* COX ∆*coxD*, ∆*coxE*, and ∆*coxF* with cysteine, glutamic acid, glycine, or nicotinic acid, concentrations equivalent to those found in SOB medium were selected (0.14 mg/ml cysteine, 3.5 mg/ml glutamate, 0.98 mg/ml glycine, 0.60 mg/ml nicotinic acid), based on elemental compositions for tryptone (ITW Reagents – 403682) and yeast extract (ITW Reagents – A1552). These nutrients were selected as a result of the rescue effect initially being observed after growing *Pb. terrae* COX in super-optimal broth (data not shown).

Calculating rates of CO uptake by *Pb. terrae* COX strains

CO uptake assays were conducted primarily according to Dawson et al. (2025). *Pb. terrae* COX wild-type, ∆*coxL-1*, ∆*coxL-2,* ∆*coxD*, ∆*coxE*, ∆*coxF*, and ∆*coxG* were grown in 10 VL55 medium with 5 mM pyruvate and 1,000 ppmv CO for 72 hours (30 °C, 150 rpm). Grown cultures were used to inoculate 400 ml VL55 medium in 2 L flasks, supplemented with 5 mM pyruvate and 1000 ppmv CO, using 3 biological replicates per

strain. *Pb. terrae* COX strains were centrifuged in 30 ml aliquots at 3000 g for 10 minutes (4 °C), washed in sterile VL55 medium, and resuspended to an OD600 of 4.0 in 1 ml VL55 medium in a 30 ml vial, sealed using a butyl rubber stopper and aluminium crimp cap. Vials were kept on ice for no longer than 10 minutes before beginning CO uptake assays.

Vials were pre-warmed for 10 minutes in a 30 °C water bath with shaking at 150 rpm, then 1000 ppmv CO was added from a 10% CO (v/v) stock. Vials were incubated for a further 1 minute before analysing 100 µl of headspace gas by gas chromatography. Headspace samples were measured every 6 minutes until 7 samples were recorded, with a final sample taken at 71 minutes following CO addition. This final timepoint was included to increase the likelihood of observing low rates of CO oxidation.

Whole genome sequencing of *Pb. terrae* COX

The genome of *Pb. terrae* COX was extracted (Dawson *et al.*, 2025)and sequenced by MicrobesNG (Birmingham, UK) using the Hybrid Sequencing service. The following methodological details were provided by MicrobesNG: sequencing libraries were generated using SQK-RBK114.96. Sequencing was performed on a GridION (Oxford Nanopore Technologies) using an R10.4.1 flowcell, with basecalling model r1041_e82_400bps_sup_variant_v4.3.0. Reads were randomly subsampled to 50X coverage using Rasusa (V 0.7.1) (Hall, 2022), and assembled using Flye (V2.9.2-b1786) (Kolmogorov *et al.*, 2019). Assemblies were polished using Medaka (V 1.11.3) (Oxford Nanopore Technologies) and the relevant model (r1041_e82_400bps_sup_variant_v4.3.0). Annotation was performed using Bakta (V 1.8.1) (Schwengers *et al.*, 2021). Taxonomic classification of the raw reads and assemblies was performed using Kraken (V 2.0.9-beta Derrick) (Wood *et al.*, 2019), and 16S identification was performed using Barrnap (available at https://github.com/tseemann/barrnap) and Sina (Bolyen *et al.*, 2019). Assemblies were analysed and visualised using Quast (Mikheenko *et al.*, 2018) and Bandage (V 0.8.1) (Wick *et al.*, 2015).

Plasmid generation

Plasmids were prepared using restriction digestion and ligation. FastDigest restriction enzymes (*XbaI*, *BamHI*, *HindIII*, *PstI*, *EcoRI)* and T4 DNA ligase were purchased from

Thermo Scientific (Waltham, MA, USA). Primers were designed using Benchling (https://benchling.com/, last accessed 12/2025) with restriction sites included (Table S2) to support assembly of mutagenesis and complementation plasmids (Table S3). Mutation primers were designed to amplify the beginning and end of the gene of interest, excluding the intervening genetic information of this gene, with the aim of assembling each “homology arm” into pK18mobsacB. PCR products were synthesised using Q5 high-fidelity DNA polymerase (NEB, Hitchin, UK) according to the manufacturer’s instructions, cleaned using HighPure PCR Product Purification Kit (Roche, Welwyn Garden City, UK), then ligated with pJET1.2 (Thermo Scientific) and propagated in *E. coli* Top10 which was transformed by heat shock. pJET constructs were isolated using a GeneJET Plasmid Miniprep Kit (Thermo Scientific), restriction digested to remove insert DNA, then ligated with pK18mobsacB (for mutation) or pBBR1MCS-2 (for complementation), with each recipient plasmid digested using the same restriction enzymes as the insert DNA. Plasmid assembly was confirmed by PCR using primers specific to the insert DNA and by restriction digestion, with separation of DNA products by 1% (w/v) agarose gel electrophoresis.

Preparation of competent cells and transformation by electroporation

Electrocompetent *Pb. terrae* COX was prepared according to Choi *et al.* (Choi *et al.*, 2006) with modifications. Briefly, 30 ml of *Pb. terrae* COX was grown in SOB medium (20 g/L tryptone, 5 g/L yeast extract, 0.5 g/L NaCl, 0.186 g/L KCl) overnight and centrifuged at 3,000 x *g* for 10 minutes at room temperature. Cell pellets were washed twice with an equal volume of room-temperature 300 mM sucrose, then resuspended in a final volume of 1 ml sucrose (300 mM). 100 µl aliquots of electrocompetent cells were transformed with 500 ng of replicable DNA (pBBR1MCS-2-based vectors) or 2 µg of non-replicable DNA (pK18mobsacB-based vectors) in sterile 2 mm gap cuvettes. Cells were transformed by exponential decay electroporation (2.5 kV, 2.5 µF, 200 Ω), mixed with 1 ml SOB medium and incubated at 30 °C for 3 hours with shaking at 150 rpm, then spread on SOB agar with 50 µg/ml kanamycin to select for plasmid maintenance. Transformation plates were incubated at 30 °C until colonies formed, typically within 3-4 days.

Plasmid acquisition was confirmed by PCR using colony biomass as the template according to Dawson *et al*. (Dawson *et al.*, 2025). Initial denaturation was held at 94 °C

for 10 minutes, then 30 cycles of amplification (95 °C 15 seconds; 63 °C 20 seconds; 72 °C with extension time held according to amplicon size), with final extension of 2-7 minutes depending on the PCR amplicon size.

Preparation of mutants using double-homologous recombination

Single-recombinants generated by electroporation were screened by PCR using colony biomass as the template, wild-type genomic DNA from *Pb. terrae* COX and the applied mutation plasmid as wild-type and mutant genotype controls, respectively, using primers specific to the gene of interest. All mutants were generated by transformation of wild-type *Pb. terrae* COX except for *Pb. terrae* COX Δ*coxL-1*:Δ*coxL-2* (form I and form II CODH), which was constructed by transforming *Pb. terrae* COX Δ*coxL-2* with pK18mobsacB:Δ*coxL-1*, a suicide plasmid derived from pK18mobsacB carrying an in-frame deletion construct of form I *coxL.* Colonies were grown overnight at 30 °C in 5 ml SOB medium with 50 µg/ml kanamycin, then spread at 10^{-5} dilution on SOB agar without antibiotic. Single-recombinants, genotypically verified again by PCR, were then grown overnight (30 °C, 150 rpm) in SOB medium without antibiotic, then serially diluted 10-fold and spread on SOB agar with 10% (w/v) sucrose to counter-select against pK18mobsacB (Schäfer *et al.*, 1994). Double-recombination events, characterised by loss of the wild-type gene copy and the pK18mobsacB plasmid backbone, were confirmed by PCR using primers specific to the mutated gene (Table S1).

Transformation of *Pb. terrae* COX by conjugation

Pb. terrae COX could not be transformed with pK18mobsacB:Δ*coxE* by electroporation. Conjugation was used instead. *E. coli* S17-1 was transformed with pK18mobsacB:Δ*coxE* by heat shock. A transformant colony was inoculated in SOB medium with 50 µg/ml kanamycin and incubated overnight at 37 °C with shaking at 170 rpm. A single colony of *Pb. terrae* COX on SOB agar was grown overnight in SOB medium without antibiotics at 30 °C with shaking at 150 rpm. *Pb. terrae* COX and *E. coli* S17-1 cultures were centrifuged at 4,000 x *g* for 10 minutes at room temperature and washed twice in an equal volume of SOB medium. Cultures were mixed at a ratio of 1:10 (*E. coli*:*Pb. terrae*) and transferred to SOB agar without antibiotic, then incubated at 30 °C overnight. Conjugated biomass was suspended in fresh SOB medium, diluted 10-fold to 10^{-4} in SOB medium, and spread on SOB agar with 50 µg/ml kanamycin and 10 µg/ml

gentamicin. Kanamycin was selected for plasmid uptake, while gentamicin selected against the donor only due to the inherent resistance of *Pb. terrae* COX (not shown). Transconjugants were genotypically verified as described for electroporated transformants.

qPCR primer design, RNA preparation, and RT-qPCR to quantify *coxL* gene transcription

qPCR primers were designed using PrimerBLAST according to Dawson *et al*. (2025) to amplify *coxL_1*, *coxL_2*, and *rpoB* as an internal reference gene. Primer specificity was determined by conventional PCR using 100 ng *Pb. terrae* COX genomic DNA as a template (94 °C for 10 minutes, 25 cycles (95 °C for 15 seconds, 63 °C for 20 seconds, 72 °C for 25 seconds), 72 °C for 30 seconds). Standards for qPCR were prepared initially by generating *coxL_1*, *coxL_2*, and *rpoB* amplicons using primers q*coxL_1F/*q*coxL_1R* (Dawson *et al.*, 2025), q*coxL_2F* (5'-GCTGGCTGATCCACAGCAT-3')/q*coxL2R* (5'-GCATCTTTTGCGGCTTTCAC-3'), and q*rpoB_F* (5'-GGAACGTGTGATCGTGTCGC-3')/q*rpoB_R* (5'-GGGATGATCCGTGCCGAGAA-3'), respectively, using Q5 high-fidelity polymerase with the above PCR cycling conditions. Amplicons were extracted from 1% (w/v) agarose gels using the Wizard SV Gel and PCR clean-up system (Promega), ligated into pJET1.2, and propagated in *E. coli* Top10 prior to plasmid DNA purification using the GeneJET Plasmid Miniprep kit. Each plasmid, containing the respective qPCR amplicon, was used to generate 10-fold dilution series from 10^8-10^1 copies/µl in sterile, nuclease-free water.

Pb. terrae COX wild-type, ∆*coxL_1*, and ∆*coxL*_2 were inoculated in sterile VL55 medium with 5 mM pyruvate and 1,000 ppmv CO, incubated for 54-72 hours at 30 °C with shaking at 150 rpm, then used to inoculate 6 x 120 ml vials per strain. 3 biological replicates were harvested after 24 hours, representing exponential growth, and the remaining 3 replicates per strain were harvested after 72 hours, representing stationary phase and the onset of CO consumption (Dawson *et al*., 2025). *Pb. terrae* COX ∆*coxL_2* was harvested after 24 hours and 54 hours. Harvesting consisted of centrifugation at 6,000 *g* for 10 minutes at room temperature, then resuspending the pelleted biomass in 1 ml Trizol reagent (Thermo Fisher Scientific). RNA was extracted according to the manufacturer's instructions. Purified RNA were immediately with Qiagen RNase-free

DNase, followed by purification using an RNeasy mini kit. This process was conducted twice, then 4 µl aliquots were removed for quality analysis (1% agarose gels), quantification (Qubit RNA High Sensitivity assay, Thermo Fisher Scientific), and confirmation of total DNA removal by PCR using primers 27F/1492R (Lane, 1991). Full RNA aliquots were immediately transferred to dry ice and stored at -80 °C until all quality analyses were performed. cDNA was generated using Superscript III reverse transcriptase (Invitrogen) using a normalised input of RNA in each reaction (23.1 ng/µl RNA), primed by Random Primers (Invitrogen). Duplicated controls were prepared for each cDNA synthesis reaction which lacked reverse transcriptase.

Fresh transformations of *Pb. terrae* COX Δ*coxL-1*:Δ*coxL-2* with pBBR1:CODH-1 or pBBR1:CODH-2 were prepared and transformants were grown in VL55 medium with 5 mM pyruvate for 24 hours. These cultures were then used to inoculate 30 ml VL55 medium supplemented with 5 mM pyruvate, 1000 ppmv CO, and RNA was extracted after 24 hours and 72 hours, as described above. cDNA synthesis was similarly carried out as described above.

qPCR was conducted using SensiFAST SYBR Hi-ROX (Meridian Bioscience) with the following reaction conditions. *coxL_1* and *coxL_2* qPCR reactions: 95 °C for 15 minutes, 30 cycles (95 °C for 1 minute, 65 °C for 30 seconds, 72 °C for 30 seconds, then reading at 82 °C for 10 seconds), followed by melt curve analysis. *rpoB* reactions were as above, but cycling was increased to 35 cycles. Reaction mastermixes were prepared according to the manufacturer's instructions with technical triplicates of 1 µl standard DNA or cDNA. qPCR was conducted using a StepOnePlus instrument (Applied Biosystems). qPCR reaction efficiencies were calculated using standard curves on each plate, generated using technical duplicates for each standard: *coxL_1*: 91.96%, *coxL_2*: 103.99%, *rpoB:* 90.96%. Standards were generated by PCR-amplifying the qPCR amplicons using Q5 high-fidelity polymerase (NEB) according to the manufacturer's instructions, isolating and purifying PCR amplicons using Wizard SV Gel and PCR Clean-Up System according to the manufacturer's instructions, then ligating these amplicons individually into pJET1.2 (Thermo Fisher Scientific) according to the manufacturer's instructions. Finally, ligated pJET vectors were used to transform *E. coli* Top10 by heat-shock, grown overnight in LB medium with 100 µg/ml carbenicillin, then plasmids were harvested by GeneJET Plasmid Miniprep kit (Thermo Fisher Scientific)

according to the manufacturer's instructions, quantified by Qubit High-Sensitivity dsDNA Kit (Thermo Fisher Scientific), and copy numbers calculated accordingly. Gene expression was determined by calculating 'quantities' relative to standard curves, normalising *coxL_1* and *coxL_2* quantities against those of *rpoB* as a housekeeping gene. The relative expression of each gene was determined after 72 hours (for wild-type and ∆*coxL_1* strains) or 54 hours (for ∆*coxL_2*) vs. 24 hours.

Bioinformatics techniques

Protein domains were predicted using BLASTp (Altschul *et al.*, 1990) against the ClusteredNR database. All alignments of amino acids or nucleotide sequences were conducted using ClustalW in MEGA 11 (Tamura *et al.*, 2021) under standard parameters determined by the software. Important residues for the activity of RcoM, His^{74}, Cys^{94}, Met^{104}, and Met^{105} were inferred through alignment with the previously described RcoM from *Pb. xenovorans* LB400 (Marvin *et al.*, 2008; Bowman *et al.*, 2016). Promoter regions between *rcoM* and *coxM* were inferred through nucleotide sequence alignments adapted from Dent *et al*. (Dent *et al.*, 2022) to identify the three discontinuous nucleotide motifs (TTC[AG][TC]GCA), labelled *d, e,* and *f*. Essential CoxL active site residues Gln^{240}, Cys^{388}, and Glu^{763} were inferred through alignment with the previously described CoxL from *Afipia carbixidovorans* OM5 (Dobbek *et al.*, 1999, 2002). DEAD-box RNA helicase motifs were inferred through alignment with previously described DEAD-box motifs from CoxD, CoxE, and CoxF from *Afipia carboxidovorans* OM5 (Pelzmann *et al.*, 2014). YHS domains were identified by amino acid alignments according to Linder (2015). Amino acids predicted to mediate interactions between CODH and CoxG were inferred through alignment with the previously described CoxG from *Mycobacterium smegmatis* mc^2155 (Kropp *et al.*, 2025).

Figures

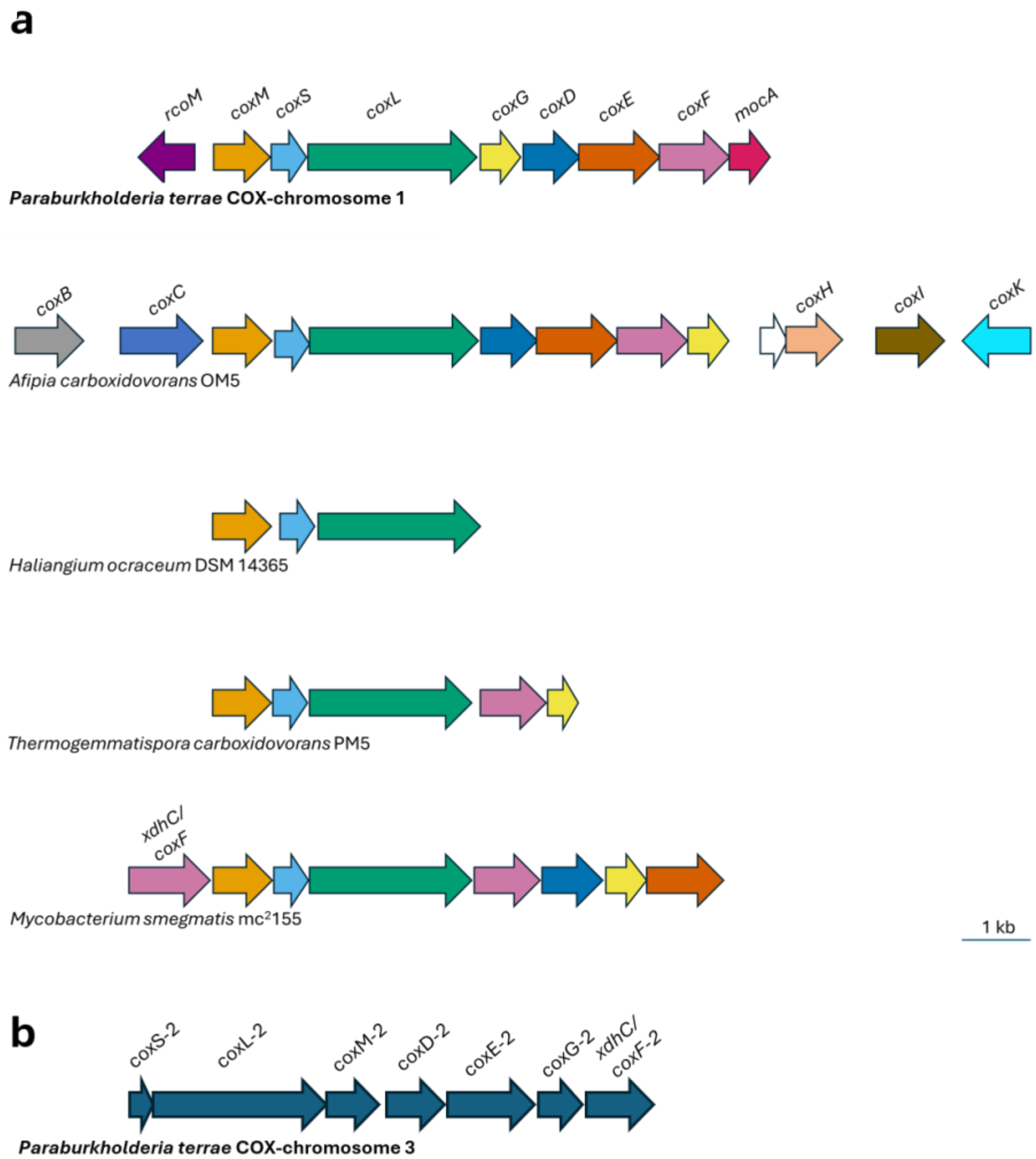

Fig. S1. a) Comparative view of form-I *cox* gene clusters from distinct CO-oxidising bacteria. b) Putative form-II *cox* gene cluster from *Pb. terrae* COX.

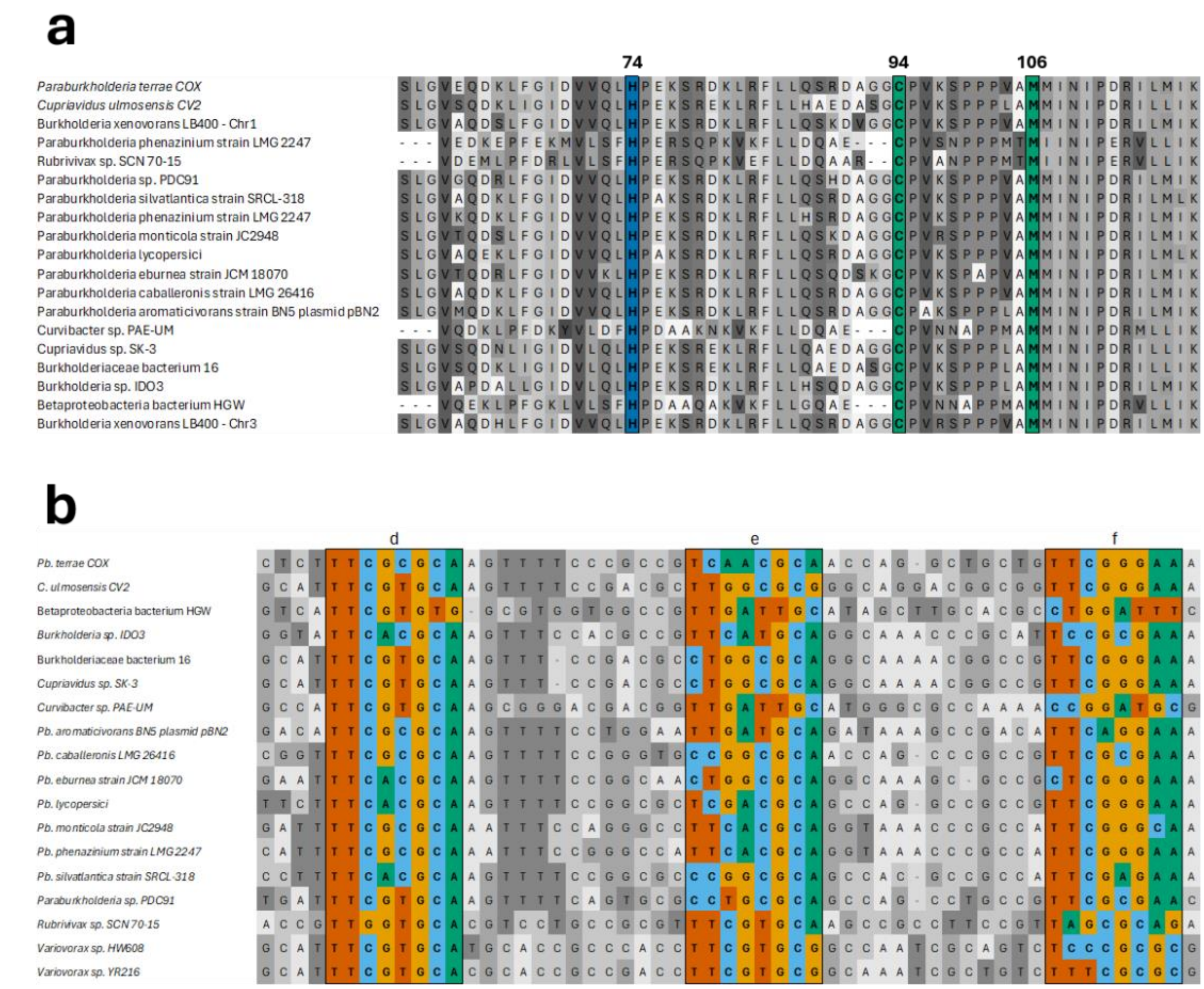

Fig. S2. a) MEGA-aligned amino acid sequences of RcoM proteins, adapted from Dent *et al*. (2022). Key residues His74, Cys94, and Met104 are highlighted in bold. b) MEGA-aligned nucleotides of *rcoM-coxM* intergenic spaces, adapted from Dent *et al.* (2022). Consensus sequences d, e, and f are highlighted in bold.

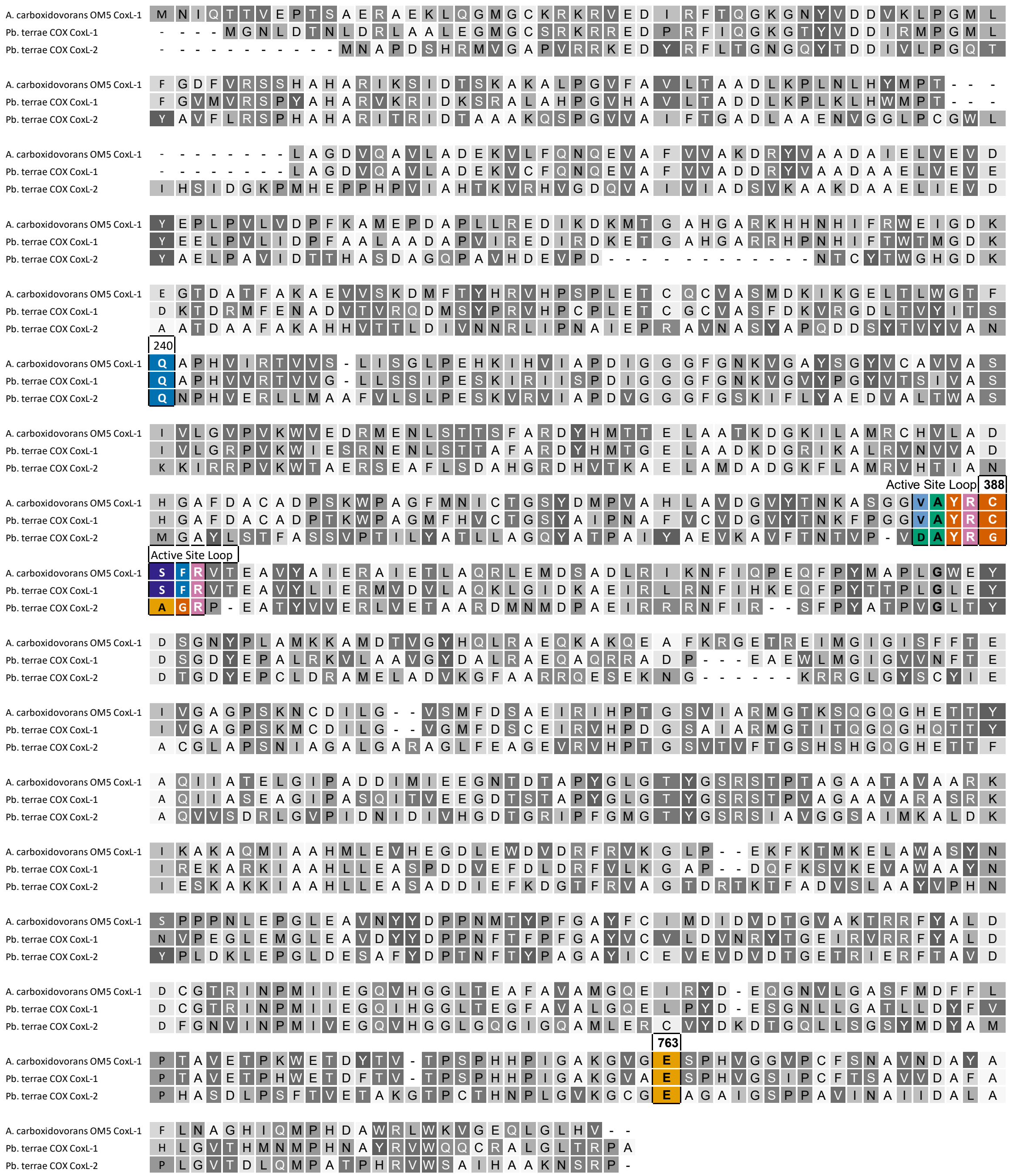

Fig. S3. MEGA-aligned amino acid residues from CoxL-1 from *A. carboxidovorans* OM5 compared to CoxL-1 and CoxL-2 from *Pb. terrae* COX. The dinucleotide-binding motif and Mo-binding residues are highlighted in bold and positions numbered.

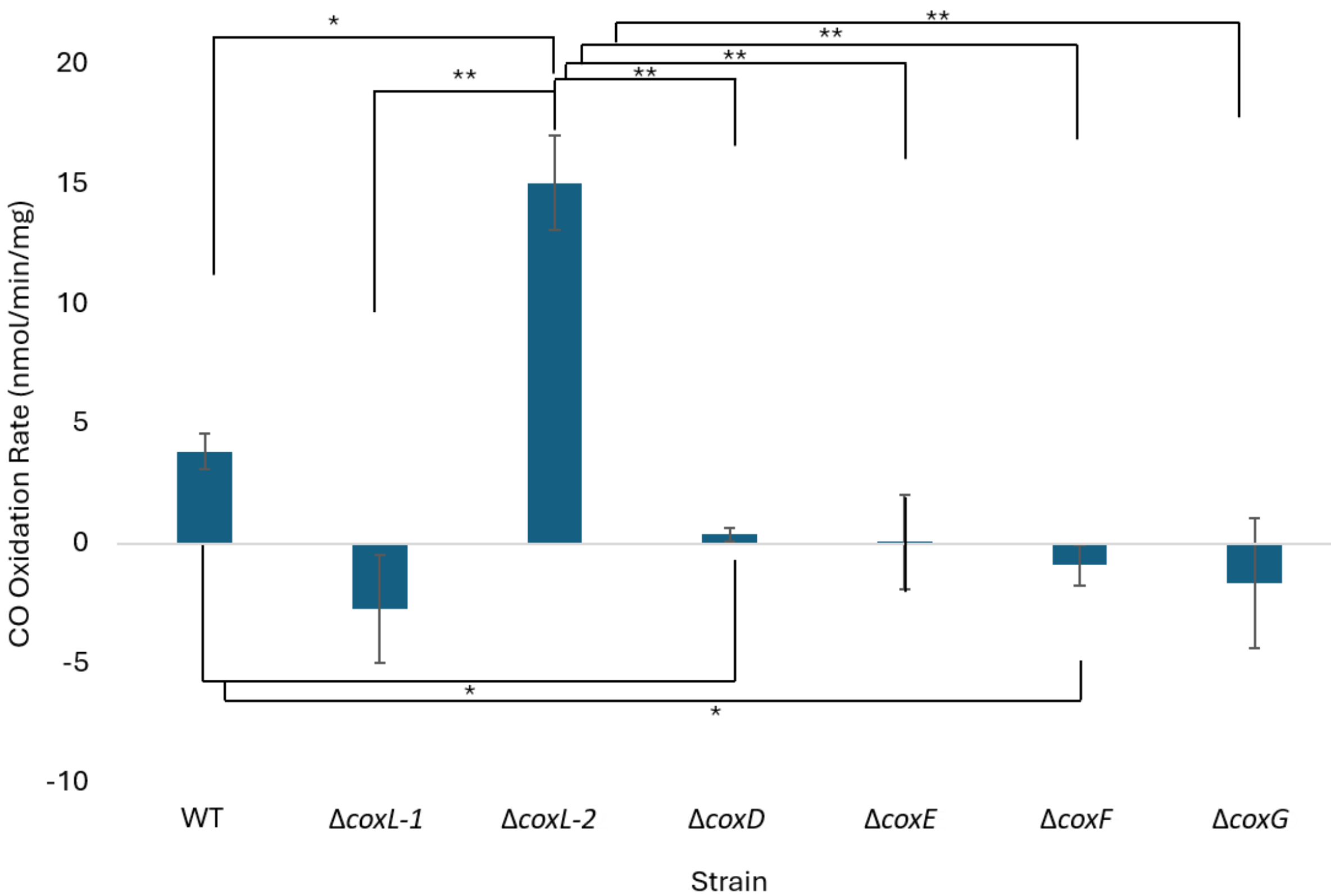

Fig. S4. Rates of CO uptake by *Pb. terrae* COX strains (nmol/min/mg). * = $p \leq 0.05$; ** = $p \leq 0.01$.

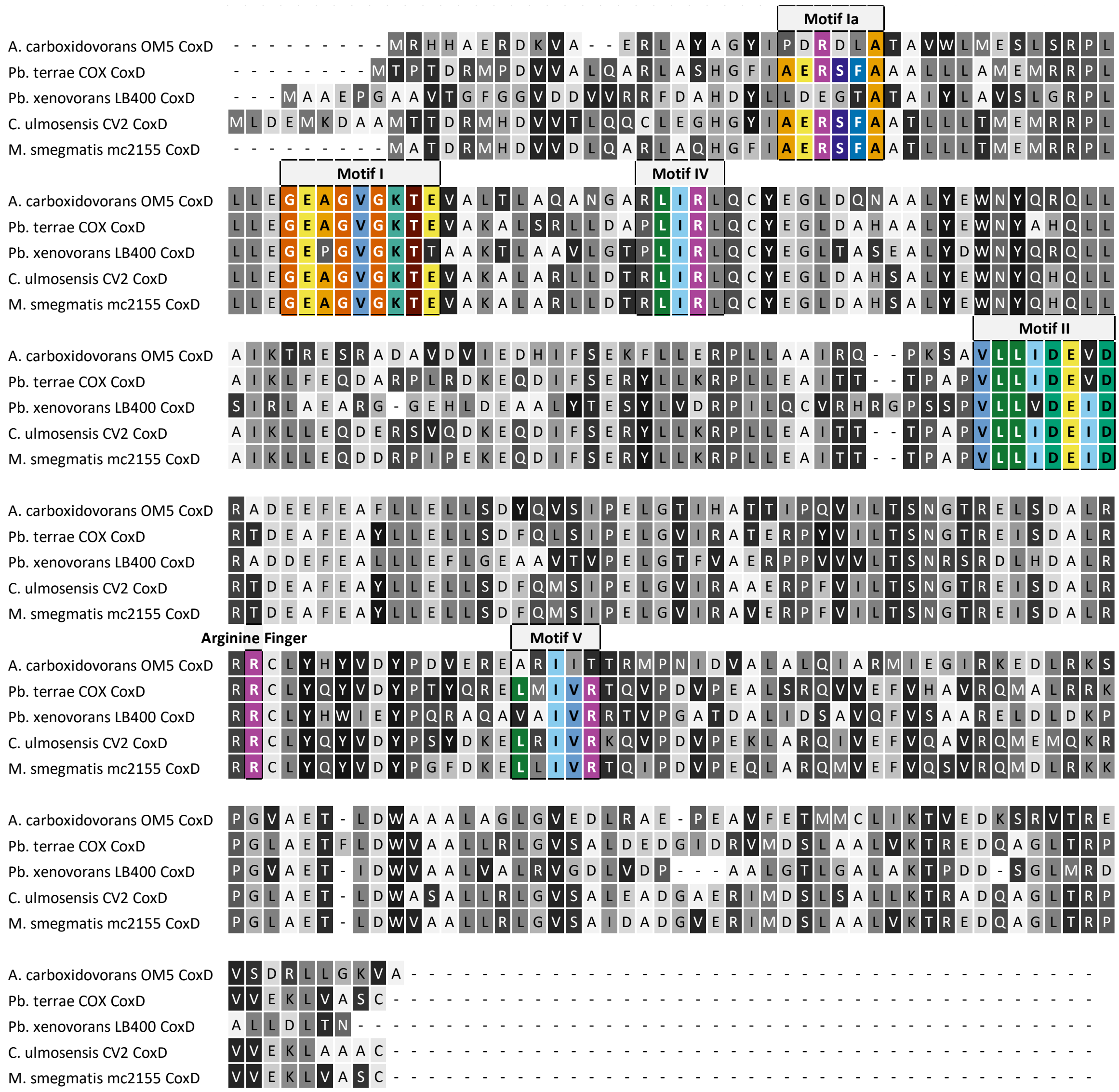

Fig. S5. MEGA-aligned amino acid residues from CoxD proteins from confirmed CO-oxidising bacteria; *A. carboxidovorans* OM5, *Pb. terrae* COX, *Pb. xenovorans* LB400, *Cupriavidus ulmosensis* CV2, and *M. smegmatis* mc^{2}155. Important motifs and domains are highlighted in bold with uniform colours.

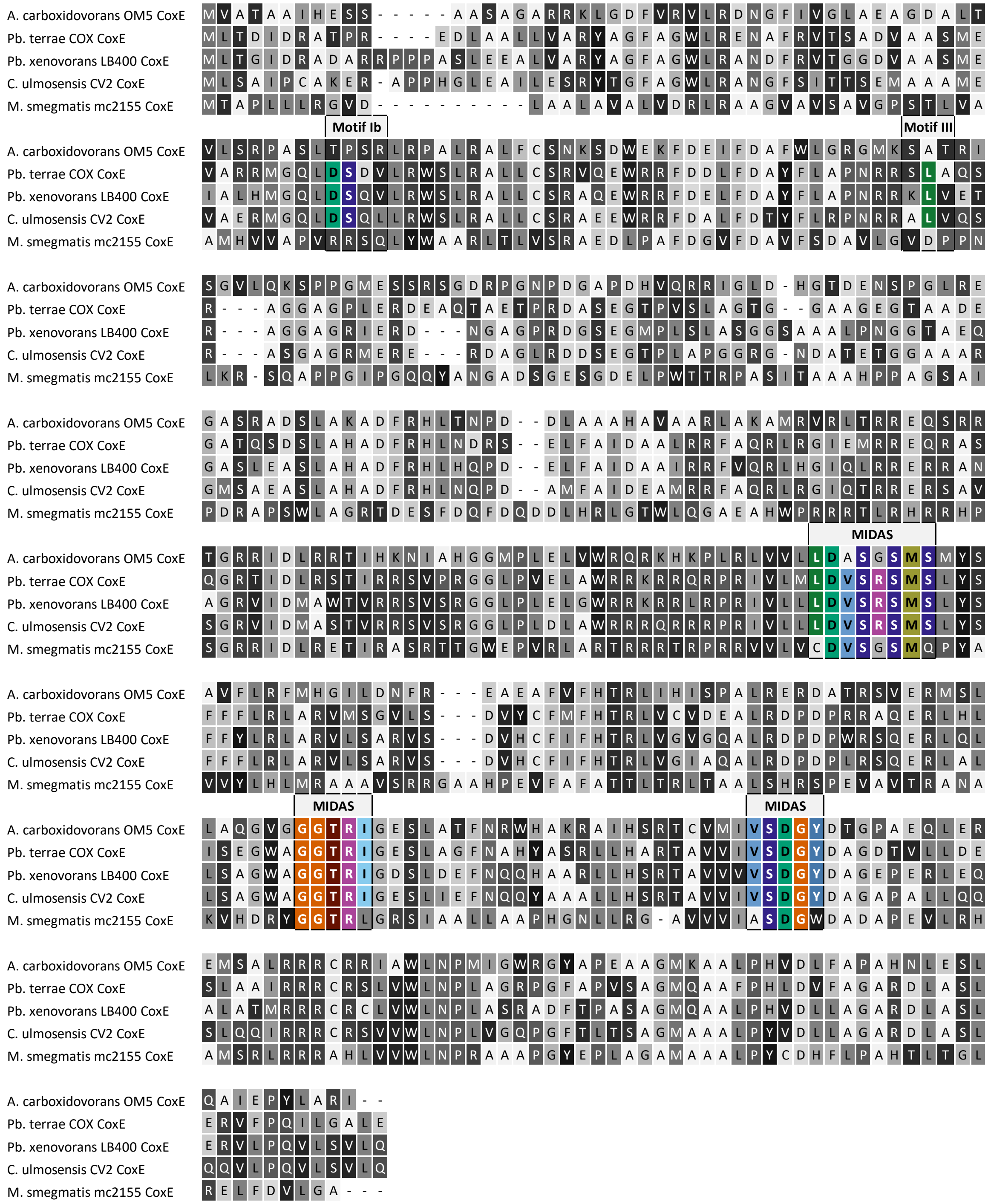

Fig. S6. MEGA-aligned amino acid residues from CoxE proteins from confirmed CO-oxidising bacteria: *A. carboxidovorans* OM5, *Pb. terrae* COX, *Pb. xenovorans* LB400, *Cupriavidus ulmosensis* CV2, and *M. smegmatis* mc^{2}155. Important motifs and domains are highlighted in bold with uniform colours.

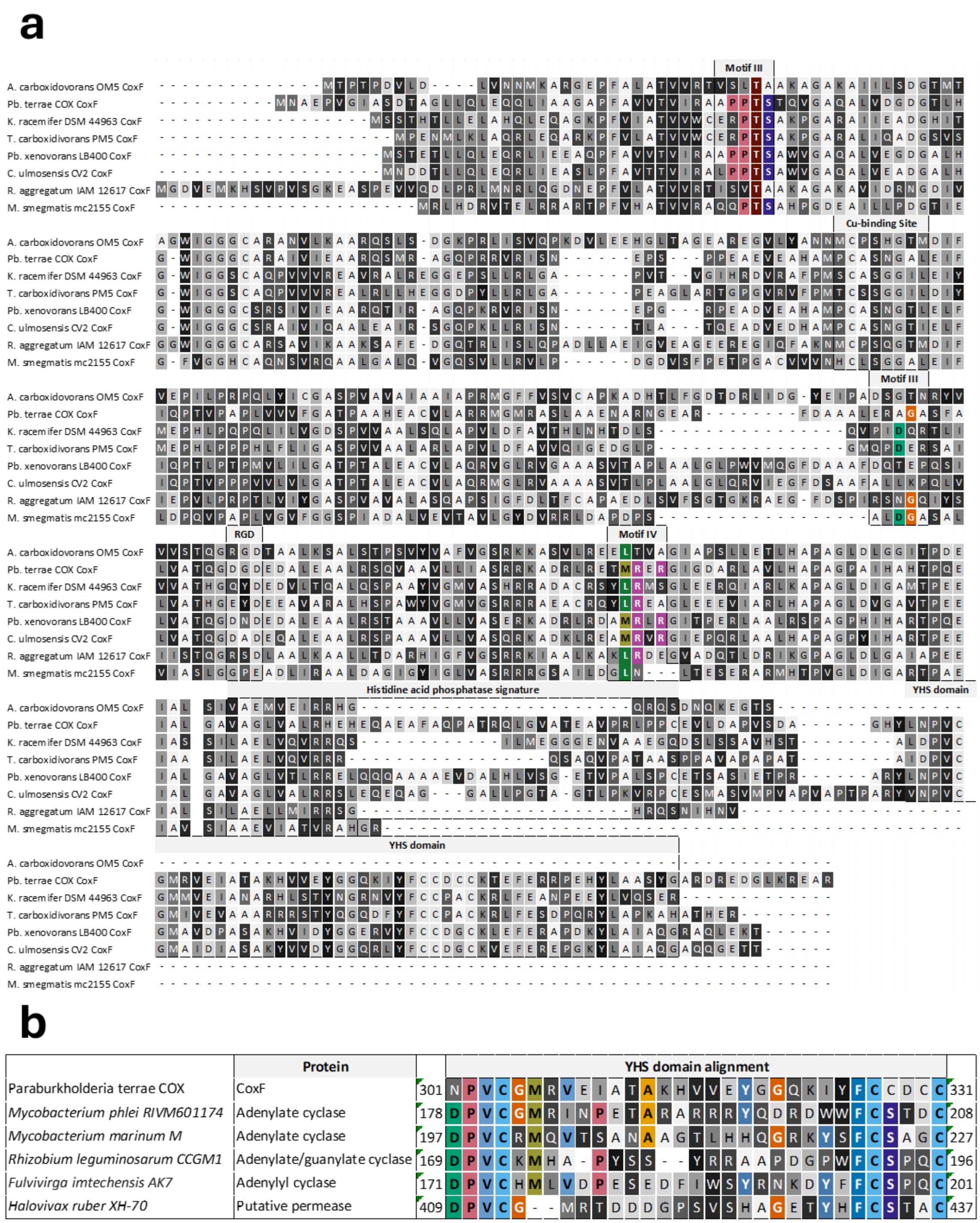

Fig. S7. a) MEGA-aligned amino acid residues from CoxF proteins from *A. carboxidovorans* OM5 and *Pb. terrae* COX. Important motifs and domains are highlighted in bold with uniform colours. b) MEGA-aligned amino acid residues from the predicted YHS domain of CoxF against YHS domains of reference proteins. This alignment was adapted from Linder (2015).

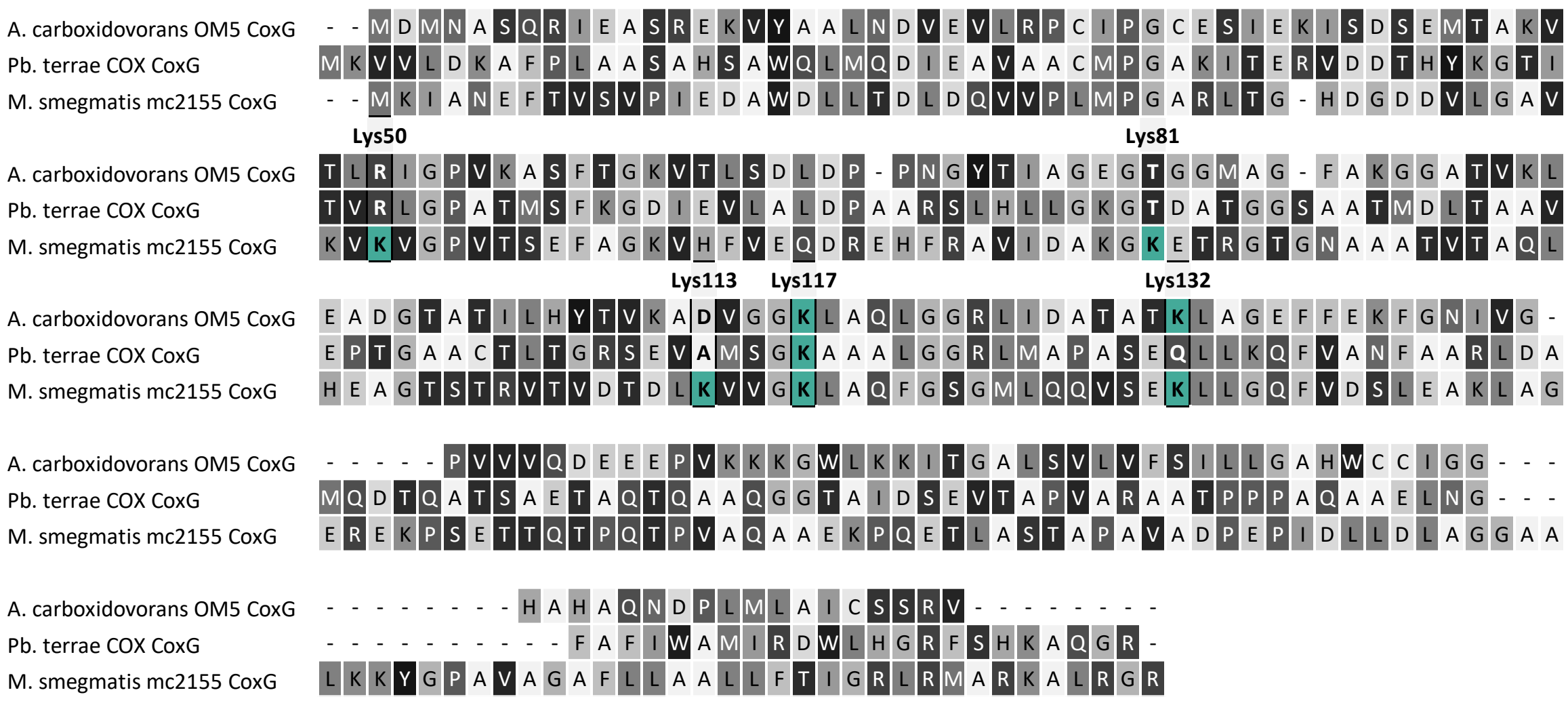

Fig. S8. MEGA-aligned amino acid sequences of CoxG from representative CO-oxidising bacteria *A. carboxidovorans* OM5, *Pb. terrae* COX, and *M. smegmatis* mc^{2}155. Conserved lysine residues (Kropp *et al.*, 2025) were highlighted in uniform colours.

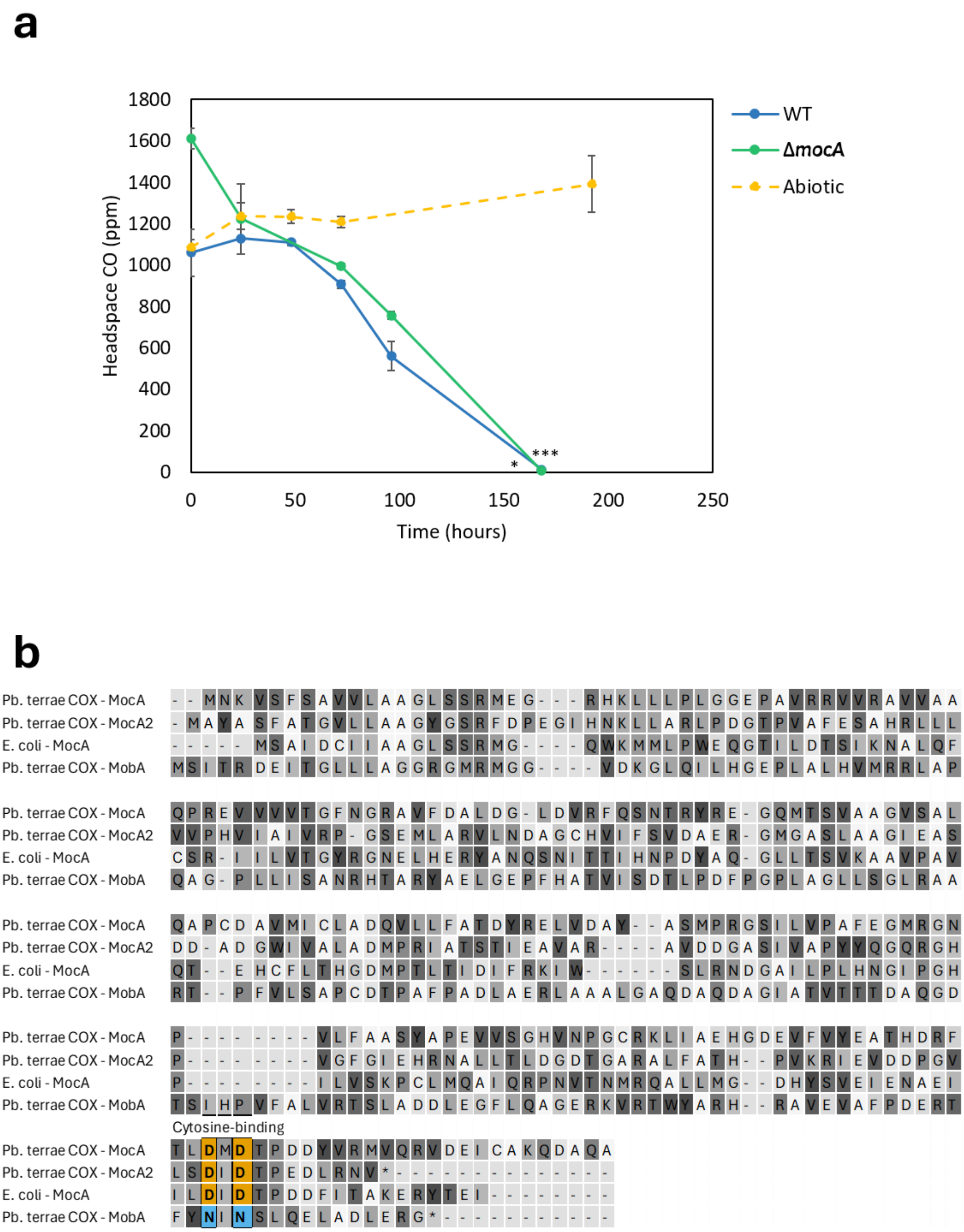

Fig. S9. Role of MocA in CO Oxidation by *Pb. terrae* COX. a) Consumption of CO by *Pb. terrae* COX Δ*mocA* compared to wild-type *Pb. terrae* COX. Error bars represent the standard error (n=3). * = $p \leq 0.05$; *** = $p \leq 0.005$. b) Aligned amino acids of MocA and MobA proteins from *Pb. terrae* COX compared to the characterised MocA from *E. coli*. Cytosine-binding residues were highlighted in uniform colours.

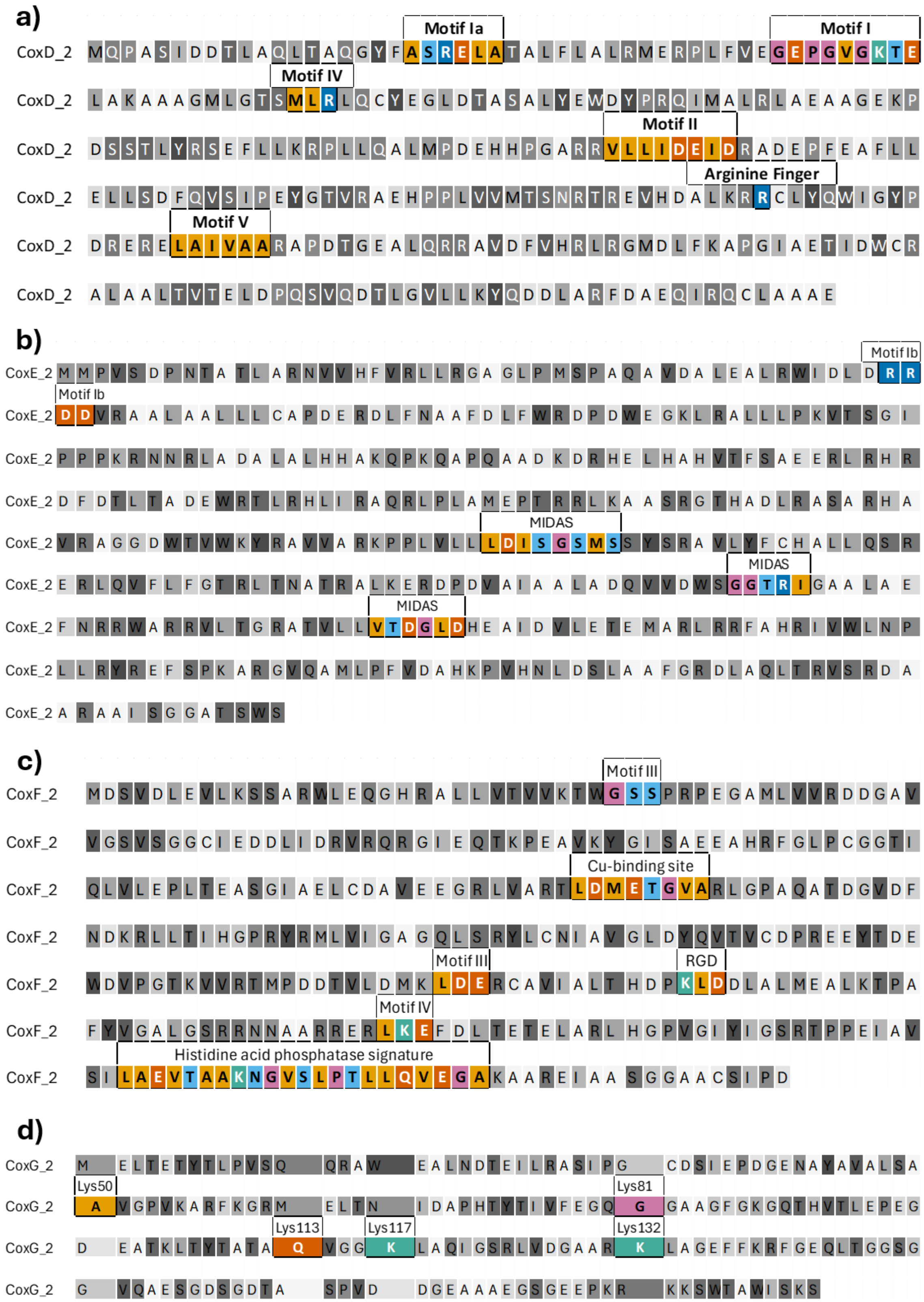

Fig. S10. Amino acids of putative CODH form II accessory proteins. a) CoxD_2, b) CoxE_2, c) CoxF_2, d) CoxG_2. Regions aligning with Cox protein motifs are highlighted based on CLUSTALW alignments.

Table S1. Strains used in this study.

Bacterium	Strain	Source
Pb. terrae COX	Wild-type	(Dawson *et al.*, 2025)
	Δ*rcoM*	This study
	Δ*coxL-1*	This study
	Δ*coxD*	This study
	Δ*coxE*	This study
	Δ*coxF*	This study
	Δ*coxG*	This study
	Δ*mocA*	This study
	Δ*coxL-2*	This study
	Δ*coxL-1:*Δ*coxL-2*	This study
E. coli	Top10	Invitrogen
	S17-1	(Simon *et al.*, 1983)

Table S2. List of primers used for mutagenesis and complementation in this study. Highlighted regions denote restriction enzyme recognition sequences.

Primer	Sequence (5'- -3')	Restriction site
	Mutagenesis	
rcoM		
rcoM-up_fwd	**GGATCC**GGTACGTCGGTCAGTAATTGG	*BamHI*
rcoM-up_rev	**TCTAGA**AGCCGGGCTGTTGAAGGAG	*XbaI*
rcoM-down_fwd	**TCTAGA**TCCAGCCGGTGCTGGAAG	*XbaI*
rcoM-down_rev	**AAGCTT**TGCCGAGGTCGATCAGATGC	*HindIII*
coxL-1		
coxL-up_fwd	**GGATCC**GCTGCTGATTCACACGCTAAGG	*BamHI*
coxL-up_rev	**TCTAGA**CGGACTGCGCACCATCAC	*XbaI*
coxL-down_fwd	**TCTAGA**TCTCGGCGTCACGCACATGAAC	*XbaI*
coxL-down_rev	**AAGCTT**CGCGACGAACTGCTTGAGCAAC	*HindIII*
coxD		
coxD-up_fwd	**CTGCAG**ATCGGAGCAGTTGCTCAAG	*PstI*
coxD-up_rev	**TCTAGA**CAGATAGCGCTCGGAGAAG	*XbaI*
coxD-down_fwd	**TCTAGA**GTGCGGCAGATGGCGTTG	*XbaI*
coxD-down_rev	**GAATTC**CGTTCCTGCCAGCGAGAC	*EcoRI*
coxE		
coxE-up_fwd	**GGATCC**GTGCGGCAGATGGCGTTG	*BamHI*
coxE-up_rev	**TCTAGA**CGTTCCTGCCAGCGAGAC	*XbaI*
coxE-down_fwd	**TCTAGA**AGCGGCTGCATCTGATCTC	*XbaI*
coxE-down_rev	**AAGCTT**CCGCGCAGCTTCGATCAC	*HindIII*

coxF		
coxF-up_fwd	**GGATCC**GTGATCGTCAGCGACGGTTATG	*BamHI*
coxF-up_rev	**TCTAGA**CGACGGCTCGTTGCTGATG	*XbaI*
coxF-down_fwd	**TCTAGA**AGCGATGCCGGGCATTATCTG	*XbaI*
coxF-down_rev	**AAGCTT**AACGCGGGCACAAGAATGGAG	*HindIII*
coxG		
coxG-up_fwd	**CTGCAG**ACGCTGCTCGACTACTTC	*PstI*
coxG-up_rev	**TCTAGA**GATCGAGCGCGAGCACTTC	*XbaI*
coxG-down_fwd	**TCTAGA**GAACGGCTTCGCCTTTATCTGG	*XbaI*
coxG-down_rev	**GAATTC**TCGACGTGAGGATCACGTAGGG	*EcoRI*
mocA		
mocA-up_fwd	**GGATCC**CGAAGCATGTCGTCGAATATGG	*BamHI*
mocA-up_rev	**TCTAGA**GAAGCCCGTCACGACAAC	*XbaI*
mocA-down_fwd	**TCTAGA**GTTGTTTGCGGCGAGCTATG	*XbaI*
mocA-down_rev	**AAGCTT**GAACGGGTGGAACGTAGGAG	*HindIII*
coxL-2		
coxL-II-up_fwd	**GGATCC**GAAATCACCACGATCGAAGG	*BamHI*
coxL-II-up_rev	**TCTAGA**GCGGCTCGTGCATCGGTTTG	*XbaI*
coxL-II-down_fwd	**TCTAGA**GGCACACCGTGTACGCACAATC	*XbaI*
coxL-II-down_rev	**AAGCT**TGGTCGGTAACGACCGTTCCATC	*HindIII*

Complementation		
**CODH-1**		
CODH-1_fwd	ATCA**AAGCTT**GTGATTCCCCGACCCTTCGAATATCACGC	*HindIII*
CODH-1_rev	ATCA**GGATCC**TCACGCAGGCCGCGTGAGACC	*BamHI*
**CODH-2**		
CODH-2_fwd	ATCA**AAGCTT**ATGGCGATCAGCATCAGTCTGACGG	*HindIII*
CODH-2_rev	ATCA**GGATCC**CTAACCGTTTGCGGCTCGCACC	*BamHI*
coxD		
*coxD*_fwd	**GGATCC**ATGACGCCCACTGACCG	*BamHI*
*coxD*_rev	**TCTAGA**TCAGCATGACGCCACCAG	*XbaI*
coxE		
*coxE*_fwd	**AAGCTT**GGCCCGTCGTCGAAAAACTGGT	*HindIII*
*coxE*_rev	**GGATCC**ACGCAATCCCCACAGGTTCAGC	*BamHI*
coxF		
*coxEF*_fwd	**GGATCC**GCGCTCGACGAAGACGGTATCG	*BamHI*
*coxEF*_rev	**TCTAGA**GCGATAGTCGGTGGCGAAGAGC	*XbaI*
coxG		
*coxG*_fwd	**AAGCTT**ATCCGCCTGCGACAGGGAGACA	*HindIII*
*coxG*_rev	**GGATCC**CATGCGGTCAGTGGGCGTCATG	*BamHI*
rcoM		
*rcoM*_fwd	**GGATCC**TATCTGCAGCGCGACGTCGTTG	*BamHI*
*rcoM*_rev	**TCTAGA**AGGTCGATCAGATGCTCGGGCT	*XbaI*
	qPCR	Source
coxL-1		
qcoxL-1_fwd	CCAGTTCAAGTCGGTCAAGGA	Dawson *et al*,. 2025
qcoxL-1_rev	CACCGAATGGGAACGTGAAG	Dawson *et al*., 2025
coxL-2		

qcoxL-2_fwd	GCTGGCTGATCCACAGCAT	*This study*
qcoxL-2_rev	GCATCTTTTGCGGCTTTCAC	*This study*
rpoB		
qrpoB_fwd	GGAACGTGTGATCGTGTCGC	*This study*
qrpoB_rev	GGGATGATCCGTGCCGAGAA	*This study*

Table S3. Plasmids used in this study.

Plasmid Name	Function	Source
	Mutagenesis	
pK18mobsacB	Suicide vector - backbone used for generation of mutation constructs.	(Schäfer *et al.*, 1994)
pk18mobsacB:Δ*rcoM*	Deletion of *rcoM* from wild-type *Pb. terrae* COX.	This study
pk18mobsacB:Δ*coxL-1*	Deletion of form-I *coxL* from wild-type *Pb. terrae* COX.	This study
pk18mobsacB:Δ*coxD*	Deletion of *coxD* from wild-type *Pb. terrae* COX.	This study
pk18mobsacB:Δ*coxE*	Deletion of *coxE* from wild-type *Pb. terrae* COX.	This study
pk18mobsacB:Δ*coxF*	Deletion of *coxF* from wild-type *Pb. terrae* COX.	This study
pk18mobsacB:Δ*coxG*	Deletion of *coxG* from wild-type *Pb. terrae* COX.	This study
pk18mobsacB:Δ*mocA*	Deletion of *mocA* from wild-type *Pb. terrae* COX.	This study
pk18mobsacB:Δ*coxL-2*	Deletion of form-II *coxL* from wild-type *Pb. terrae* COX.	This study
	Complementation	
pBBR1MCS-2	Broad host-range vector, expresses cloned genes under the control of a derepressed *lac* promoter	(Kovach *et al.*, 1995)
pBBR:*rcoM*	Complementation of Δ*rcoM* phenotype by expression of wild-type *rcoM* from pBBR1MCS-2 promoter.	This study
pBBR:CODH-1	Complementation of Δ*coxL* phenotype by expression of wild-type *coxMSL* from pBBR1MCS-2 promoter.	This study

pBBR:*coxD*	Complementation of Δ*coxD* phenotype by expression of wild-type *coxD* from pBBR1MCS-2 promoter.	This study
pBBR:*coxE*	Complementation of Δ*coxL* phenotype by expression of wild-type *coxE* from pBBR1MCS-2 promoter.	This study
pBBR:*coxEF*	Complementation of Δ*coxF* phenotype by expression of wild-type *coxEF* from pBBR1MCS-2 promoter.	This study
pBBR:*coxG*	Complementation of Δ*coxG* phenotype by expression of wild-type *coxG* from pBBR1MCS-2 promoter.	This study
pBBR:CODH-2	Complementation of Δ*coxL-I*:Δ*coxL-II* phenotype by expression of wild-type *coxMSL-II* from pBBR1MCS-2 promoter.	This study
qPCR		
pJET:*coxL-1*	pJET1.2 plasmid containing the qPCR-amplified fragment of *coxL-1* generated by primers q*coxL-1_fwd/qcoxL-1_rev.*	This study
pJET:*coxL-2*	pJET1.2 plasmid containing the qPCR-amplified fragment of *coxL-2* generated by primers q*coxL-2_fwd/qcoxL-2_rev.*	This study
pJET:*rpoB*	pJET1.2 plasmid containing the qPCR-amplified fragment of *rpoB* generated by primers q*rpoB_fwd/qrpoB_rev.*	This study

Table S4. Genome information for *Pb. terrae* COX.

	***Pb. terrae* COX**			
# Contigs	4			
Total Length (bp)	10,508,017			
Total GC (%)	62			
Structure/Plasmid no.	1	2	3	4
Topology	Circular	Circular	Circular	Circular
Size (bp)	3,201,017	2,163,235	3,985,594	1,096,568
Contig GC	62.0	62.1	62.4	60.0
# Subsystems	126	70	283	41
# Coding Sequences	3,155	2,077	3,755	1,239
# RNAs	11	0	68	7
Pseudogenes	6	0	4	2
N50	3,201,017			
N75	2,163,235			
L50	2			
L75	3			
Completeness (% - CheckM)	99.7			
Contamination (% - CheckM)	2.45			
#Ns per 100 kbp	0			
Key Gene Clusters	CODH		CODH_2	

SI References

Altschul, S.F., Gish, W., Miller, W., Myers, E.W., and Lipman, D.J. (1990) Basic local alignment search tool. *J Mol Biol* **215**: 403–410.

Bolyen, E., Rideout, J.R., Dillon, M.R., Bokulich, N.A., Abnet, C.C., Al-Ghalith, G.A., et al. (2019) Reproducible, interactive, scalable and extensible microbiome data science using QIIME 2. *Nat Biotechnol* **37**: 852–857.

Bowman, H.E., Dent, M.R., and Burstyn, J.N. (2016) Met104 is the CO-replaceable ligand at Fe(II) heme in the CO-sensing transcription factor BxRcoM-1. *Journal of Biological Inorganic Chemistry* **21**: 559–569.

Choi, K.H., Kumar, A., and Schweizer, H.P. (2006) A 10-min method for preparation of highly electrocompetent *Pseudomonas aeruginosa* cells: Application for DNA fragment transfer between chromosomes and plasmid transformation. *J Microbiol Methods* **64**: 391–397.

Dawson, R.A., Fantom, N., Martin-Pozas, T., Aguila, P., King, G.M., and Hernández, M. (2025) Carbon monoxide-oxidising Pseudomonadota on volcanic deposits. *Environ Microbiome* **20**:.

Dent, M.R., Roberts, M.G., Bowman, H.E., Weaver, B.R., McCaslin, D.R., and Burstyn, J.N. (2022) Quaternary structure and deoxyribonucleic acid-binding properties of the heme-dependent, CO-sensing transcriptional regulator PxRcoM. *Biochemistry* **61**: 678–688.

Dobbek, H., Gremer, L., Kiefersauer, R., Huber, R., and Meyer, O. (2002) Catalysis at a dinuclear [CuSMo(=O)OH] cluster in a CO dehydrogenase resolved at 1.1-Å resolution. *Proc Natl Acad Sci U S A* **99**: 15971–15976.

Dobbek, H., Gremer, L., Meyer, O., and Huber, R. (1999) Crystal structure and mechanism of CO dehydrogenase, a molybdo iron-sulfur flavoprotein containing *S*-selanylcysteine. *Proc Natl Acad Sci U S A* **96**: 8884–8889.

Hall, M.B. (2022) Rasusa: Randomly subsample sequencing reads to a specified coverage. *J Open Source Softw* **7**: 3941.

Kolmogorov, M., Yuan, J., Lin, Y., and Pevzner, P.A. (2019) Assembly of long, error-prone reads using repeat graphs. *Nat Biotechnol* **37**: 540–546.

Kovach, M.E., Elzer, P.H., Hill, D.S., Robertson, G.T., Farris, M.A., Roop, R.M., and Peterson, K.M. (1995) Four new derivatives of the broad-host-range cloning vector PBBR1MCS, carrying different antibiotic-resistance cassettes. *Gene* **166**: 175–176.

Kropp, A., Gillett, D.L., Venugopal, H., Gonzálvez, M.A., Lingford, J.P., Jain, S., et al. (2025) Quinone extraction drives atmospheric carbon monoxide oxidation in bacteria. *Nat Chem Biol* **21**: 1058–1068.

Lane, D.J. (1991) 16S/23S rRNA sequencing. In *Nucleic acid techniques in bacterial systematics*. Stackebrant, E. and Goodfellow, M. (eds). New York: John Wiley & Sons, pp. 115-175. doi: 10.1002/jobm.3620310616.

Linder, J.U. (2015) The YHS-domain of an adenylyl cyclase from *Mycobacterium phlei* is a probable copper-sensor module. *PLoS One* **10**:.

Marvin, K.A., Kerby, R.L., Youn, H., Roberts, G.P., and Burstyn, J.N. (2008) The transcription regulator RcoM-2 from *Burkholderia xenovorans* is a cysteine-ligated hemoprotein that undergoes a redox-mediated ligand switch. *Biochemistry* **47**: 9016–9028.

Mikheenko, A., Prjibelski, A., Saveliev, V., Antipov, D., and Gurevich, A. (2018) Versatile genome assembly evaluation with QUAST-LG. *Bioinformatics* **34**: i142–i150.

Pelzmann, A.M., Mickoleit, F., and Meyer, O. (2014) Insights into the posttranslational assembly of the Mo-, S- and Cu-containing cluster in the active site of CO

dehydrogenase of *Oligotropha carboxidovorans*. *Journal of Biological Inorganic Chemistry* **19**: 1399–1414.
Schäfer, A., Tauch, A., Jäger, W., Kalinowski, J.J., Thierbach, G., and Pühler, A. (1994) Small mobilizable multi-purpose cloning vectors derived from the *Escherichia coli* plasmids pK18 and pK19: selection of defined deletions in the chromosome of *Corynebacterium glutamicum*. *Gene* **145**: 69–73.
Schwengers, O., Jelonek, L., Dieckmann, M.A., Beyvers, S., Blom, J., and Goesmann, A. (2021) Bakta: rapid and standardized annotation of bacterial genomes via alignment-free sequence identification. *Microb Genom* **7**:.
Simon, R., Priefer, U., and Pühler, a. (1983) A broad host range mobilization system for *in vivo* genetic engineering: transposon mutagenesis in gram negative bacteria. *Bio/Technology* **1**: 784–791.
Tamura, K., Stecher, G., and Kumar, S. (2021) Molecular evolutionary genetics analysis version 11. *Mol Biol Evol* **38**: 3022–3027.
Wick, R.R., Schultz, M.B., Zobel, J., and Holt, K.E. (2015) Bandage: interactive visualization of de novo genome assemblies. *Bioinformatics* **31**: 3350–3352.
Wood, D.E., Lu, J., and Langmead, B. (2019) Improved metagenomic analysis with Kraken 2. *Genome Biol* **20**: 257.
